# Chemoinformatics analysis of *Mangifera indica* leaves extracted phytochemicals as potential EGFR kinase modulators

**DOI:** 10.3389/fchem.2025.1524384

**Published:** 2025-03-24

**Authors:** Md. Abdullah Al Mashud, Ajoy Kumer, Ismat Jahan, Md. Mehedi Hasan Somrat, Md. Enamul Kabir Talukder, Md. Mashiar Rahman, A. F. M. Shahab Uddin, Md. Harun-Or-Rashid, Md. Mizanur Rahman, Mohammad Harun-Ur-Rashid, Gamal A. Shazly, Youssouf Ali Younous

**Affiliations:** ^1^ Biophysics and Biomedicine Research Lab, Department of Electrical and Electronic Engineering, Islamic University, Kushtia, Bangladesh; ^2^ Computational Bio-Info Lab, Research and Development Center for Sustainability, Scientific Foundation for Cancer Research, Kushtia, Bangladesh; ^3^ Center for Global Health Research, Saveetha Medical College and Hospitals, Saveetha Institute of Medical and Technical Sciences, Chennai, Tamil Nadu, India; ^4^ Department of Chemistry, College of Arts and Sciences, IUBAT-International University of Business Agriculture and Technology, Dhaka, Bangladesh; ^5^ Molecular and Cellular Biology Laboratory, Department of Genetic Engineering and Biotechnology, Jashore University of Science and Technology, Jashore, Bangladesh; ^6^ Department of Computer Science and Engineering, Jashore University of Science and Technology, Jashore, Bangladesh; ^7^ School of Engineering, Macquarie University Sydney, Sydney, NSW, Australia; ^8^ Computational Biology Research Laboratory, Department of Pharmacy, Faculty of Health and Life Sciences, Daffodil International University, Dhaka, Bangladesh; ^9^ Department of Mathematics, Islamic University, Kushtia, Bangladesh; ^10^ Department of Pharmaceutics, College of Pharmacy, King Saud University, Riyadh, Saudi Arabia; ^11^ Evangelical College BP 1200, N’Djamena, Chad

**Keywords:** breast cancer, ADMET, frontier molecular orbitals, DFT, molecular dynamic simulation, 3w32 protein. −5.668

## Abstract

Breast cancer, being among the most frequent and fatal cancers in women, is an enormous issue globally. The critical requirement for novel treatment methods is underscored by its high mortality rate and relentless advancement. Even though breast cancer is one of the world’s most common causes of death, the therapeutic avenue is still limited. The aim of this work is to investigate the potential inhibitory effects of specific compounds present in leaf extract from *Mangifera indica* on the growth of drug-resistant breast cancer protease PDB ID 3w32. The chemical compounds present in *Mangifera indica* leaves were used to analyze using molecular modeling techniques, such as molecular docking, molecular dynamics (MD) simulations, quantum mechanics (QM) calculations, and the Absorption, Distribution, Metabolism, Excretion, and Toxicity (ADMET) method, in order to examine three key chemical constituents: quercetin (08), catechin (09), and elagic acid (10). The ligands undergo extensive testing to figure out how effective they are against the 3w32-overexpressing breast cancer protein. Quantum calculations retaining HOMO-LUMO analysis might identify important characteristics of molecules, such as chemical potential, electronegativity, hardness, softness, and orbital energy gaps. According to the molecular docking inquiry, ligands 08, 09, and 10 are strong candidates with strong binding affinity for the breast cancer protein that overexpresses 3w32. The protein binding site stability of the chosen natural ligands was verified by MD simulation. These three ligands not only surpass the efficacy of the FDA-approved treatment, but also fulfill the requirements for a possible new inhibitor of breast cancer.

## Introduction

Breast cancer is a life-threatening problem around the world particularly women. It is common and ranks as the second-most deadly cancer among all cancers (Sun et al., 2017; Kolak et al., 2017) as well as widely viewed as a disease that affects older women and is thought to be relatively uncommon in younger women (Azim and Partridge, 2014). The risk factors of Breast cancer include sex, age, family history, reproductive variables (late menopause, early menarche, low parity, first pregnancy at a late age, nursing, abortion, number of live births, and so on), estrogen, and lifestyle (Rojas and Stuckey, 2016). Benign breast tumors began as ductal hyperproliferation and were later transformed into malignant or metastatic breast tumors using mutagens. Some genes, such as BRCA1, BRCA2, HER2, Epidermal Growth Factor Receptor (EGFR), c-Myc, Ras, and others, are also linked to breast cancer (Sun et al., 2017).

Treatment for breast cancer is determined by its stage, biomarkers, and histology. Chemotherapy, radiation therapy, surgery, endocrine therapy, and neoadjuvant or adjuvant chemotherapy are some of the treatments available (Hortobagyi, 1998; Fisusi and Akala, 2019). Some medicines, such as capecitabine, gemcitabine, vinorelbine, taxane, anthracycline, methotrexate, mitomycin C, docetaxel, and cisplatin, are used in various combinations to treat breast cancer and are administered by nanoparticles due to their diverse properties as drug delivery vehicles (Fisusi and Akala, 2019; Tran et al., 2020). However, these treatments are costly, can lead to further post-treatment problems, and cancer recurrence is common. Keeping these dangers in mind, researchers are currently working to produce novel medications from natural sources that will cure breast cancer more effectively while also assuring patient safety.

*Mangifera indica* is a popular fruit plant in the Anacardiaceae family, and extensive research has been conducted on its leaves due to its numerous health advantages. *Mangifera indica L*. leaves contain a variety of phytochemicals, including gallic acid, protocatechuic acid, shikimic acid, mangiferin, homomangiferin, and quercetin. These leaves are also high in proteins, vitamins, and minerals, and they have antimicrobial, antioxidant, anti-diabetic, anti-cancer, lipid-lowering, hepatoprotective, anti-obesity, and anti-diarrheal effects (Kumar et al., 2021; Mirza et al., 2021). It is already being researched as a potential treatment for breast cancer. Researchers discovered that the phytochemicals in this plant extract can effectively suppress breast cancer cell growth, proliferation, invasion, and migration while also initiating cell cycle arrest and death (Rasul et al., 2021; Yap et al., 2021a).

We applied Computer-aided drug design (CADD) to investigate the anticancer properties of the phytochemical presents in this plant. This *in silico* approaches provide dynamics in overall drug design and development by reducing costs, time, and laboratory equipment (Macalino et al., 2015; Yu and MacKerell, 2017). The major goal of this work is to identify prospective therapeutic candidates from phytochemicals found in *Mangifera indica L*. leaves against particularly sensitive breast cancer proteins.

## Materials and methods

### Ligand’s profiling and optimization

Only ligands that meet particular criteria, such as being in the correct tautomer and ionization states and having the correct bond ordering, can be used in virtual screening (Madhavi Sastry et al., 2013). The Gaussian version 09 approach and realistic density functional theory (DFT) methodologies were employed to accomplish substantial atomic enlargement (Hosen et al., 2021). We employed a modified version of the Gaussian code with modifications to its polarization capability premise set (DNP), B3LYP, and Gaussian version 09 capabilities to obtain the highest achievable accuracy (Mohapatra et al., 2021). The files that represent electron negativity, electron partiality, energy gap, synthetic potential, hardness, delicate quality, and electrophilicity were solved using the criteria listed in the order (Equations 1–8). Subatomic limit orbital charts (HOMO and LUMO) were then calculate using mathematical procedures of given equations. The modified particle was then stored in a PDB file.

$$E_{\text{gap}}=\left({\;E}_{\text{LUMO}}-E_{\text{HOMO}}\right)$$ (1)

$$I=-E_{\text{LUMO}}$$ (2)

$$A=-E_{\text{HOMO}}$$ (3)

$$\left(\chi \right)=\frac{I+A}{2}$$ (4)

$$\left(\omega \right)=\frac{\mu^{2}}{2\eta }$$ (5)

$$\left(µ\right)=-\frac{I+A}{2}$$ (6)

$$\left(\eta \right)=\frac{I-A}{2}$$ (7)

$$\left(S\right)=\frac{1}{\eta }$$ (8)

### Prediction of activity spectra (PASS) assessment

The PASS Online resource is freely available on the internet (http://www.way2drug.com/passonline). This tool seeks to predict the biological activity spectra of organic compounds for over 4000 different types of biological activity using their structural formulae (Poroikov et al., 2019). Over 95% of the time, it properly predicts the outcome. Researchers examined the structure-activity relationships in the training set to create the forecast. This set contains information on the structures and biological functions of approximately 300,000 chemical molecules. We examine the advantages and disadvantages of this strategy. There is information available on how to interpret the forecast’s findings (Druzhilovskiy et al., 2016). The PASS Online website has real-world applications that prioritize chemical synthesis and biological testing based on prediction findings. New pharmacological medications are being created, and PASS Online is expected to play a growing role as a multidisciplinary academic research center in this area (Siddikey et al., 2022).

### Pharmacokinetics properties assessment

Pharmacokinetics refers to the mathematical study of the ADME characteristics of a drug in relation to its dose and duration. The computational drug design and development process helps optimize a molecular candidate into a viable treatment by assessing pharmacokinetic features early on (Zhou et al., 2016). Therefore, the pharmacokinetic parameters of the selected drugs were determined using the Swiss-ADME server (http://www.swissadme.ch/index.php) (Daina et al., 2017). By utilizing the server, one can observe and anticipate the drug’s various pharmacokinetic and pharmacodynamics features.

### Protein retrieved and preparation

A number of different proteins contribute to the development of breast cancer in females. Proteins with PDB IDs 3W32 were chosen as breast cancer susceptibility proteins after careful consideration of literature, methodologies, resolution, and organisms utilized for protein isolation. The choosing factors of the protein PBD ID 3w32 is depicted in the Supplementary Table S1. Crystal structures of these proteins were sourced from the Protein Data Bank (PDB) maintained by the RCSB (https://www.rcsb.org) (Burley et al., 2023). In order to remove water molecules and protein ligands, proteins were purified using the PYMOL software (version 2.4.1) before protein production. The website CASTp (http://sts.bioe.uic.edu/castp/index.html?2was) was utilized to gather the active site residues of the target proteins. Information on the proteins that were produced is shown in Table 1.

**TABLE 1:** Information of protein’s selected for breast cancer.

Properties	3W32
Method	X-ray diffraction
Resolution	1.80 Å
Organism	*Homo sapiens*
Active Site Residues	LEU718, GLY719, SER720, GLY721, ALA722, PHE723, GLY724, VAL726, ALA743, LYS745, MET766, VAL769, ASP770, ASN771, VAL774, CYS775, ARG776, LEU777, LEU778, THR790, GLN791, LEU792, MET793, GLY796, CYS797, LEU799, ASP800, ASP837, ARG841, ASN842, LEU844, LYS852, THR854, ASP855, PHE856, LEU858, PRO877, PHE997, LEU1001, ALA1013, ASP1014, LEU1017

### Binding site identification and receptor grid generation

**FIGURE 1:**
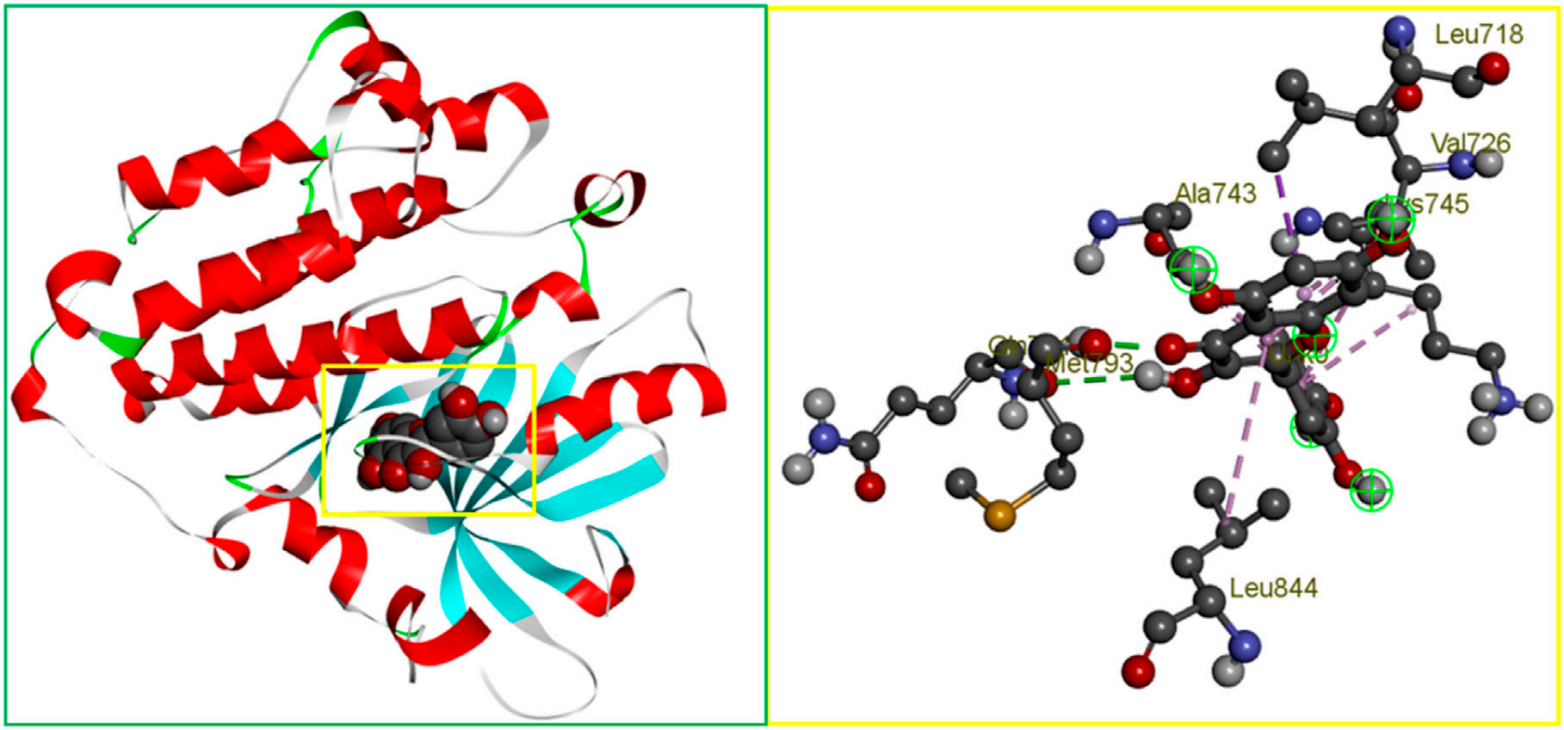
The binding site position of breast cancer identified from the protein–ligand complex (PDB ID: 3w32) structure. Ball shape 3D representation of the binding site with the grid box shown on the left side in the figure, where 2D binding site position has also been represented on the right side of the figure.

In protein-ligand interactions, binding sites can be found by looking for well-known pockets. The protein’s binding site was examined using BIOVIA Discovery Studio Visualizer v19.1 (BIOVIA) following a PDB search for the known and experimentally verified protein structure in complex with the ligand (PDB ID: 3w32). As shown in Figure 1, the binding site obtained from the complex structure was used in the receptor grid construction during the molecular docking using the PyRx virtual screening tool.

### Molecular docking simulation

To find the best hit candidates against the needed protein, a molecular docking simulation was performed using the PyRx program (Dallakyan and Olson, 2015). PyRx is a free, free and open-source virtual screening implementation that also includes the docking wizards AutoDock 4 and Vina. It may search a big database of molecules for a specific macromolecule with a medical application. We used the AutoDock Vina wizard with Pyrex’s default settings to simulate molecular docking. To begin, we used Pyrex’s conjugate gradient approach to reduce the energy of the chosen ligands in the Merc molecular force field (mmff94 force field). The final phase of docking was to convert these energy to PDBQT format. Autodocking was performed with grid box dimensions of 58.5633 X, 53.9165 Y, and 66.1292 Z, with center X, Y, and Z values of 18.8128, 25.7483, and 14.2748, respectively. All of the ligands and proteins had their surfaces covered with a grid box.

### Quantum mechanics (QM) calculation

The conformation of the ligand to the protein binding site is the most important factor in determining a potential active conformation, binding affinity, and strain discipline related to the binding mechanism. Structure optimization and least energy conformation methods based on the solution phase and the same gas-phase energy can be used to accomplish this kind of binding. Because of the metal ions, the ligand-protein combination does not conform to the expectations of classical molecular mechanics (MM) (Bronowska, 2011). The development of scoring functions that may describe electronic structure, electronic transitions, and system-specific charges in a molecular system reaction has recently been greatly aided by QM-based calculations. Eighty to ninety percent of modern quantum mechanics (QM) calculations employ DFT. For this study, DFT quantum mechanical computations on a subset of molecules were required. We started by finding compounds with the best possible bond lengths, angles, and dihedral angles. The DFT of the compounds was then calculated using Gaussian version 09 (Abdelrhman et al., 2021). The B3LYP functional set was utilized by.

DFT. The DFT needed the 6311G basis set to describe the molecule’s electronic wave functions.

### Molecular dynamics simulation

The degree of stability of the chosen candidate compounds when bound to the active site cavity of the target protein was assessed by applying molecular dynamics (MD) simulations on the complex structure for a duration of 100 nanoseconds. This technique was done in order to assess whether the binding was secure. A molecular dynamics (MD) simulation of the complicated structure was performed in the Schrodinger research edition using the ‘Desmond v3.6 Program’ (Bharadwaj et al., 2021). The Linux operating system was used to conduct this simulation. This process has to be followed in order to ascertain whether thermodynamic stability is present in the receptor-ligand pair. Using a pre-set TIP3P water model and making sure the volume didn't change during the procedure solved the issue. By positioning an orthorhombic periodic box shape at a distance of 10 s on either side of the border, this was possible. It was found that the right ions, such as Na+ and Cl-, were selected and then randomly distributed throughout the solvated system in order to electrically neutralize it. The concentration of salt was 0.15 M. The Desmond module defined a procedure for reducing and relaxing the ligand and protein after they had come together to form the solvated system. The characteristics of the OPLS-2005 force field were used in this technique. The NPT ensemble was kept at a temperature of 300 K and an atmospheric pressure of one (1.01325 bar) by using the Nose-Hoover temperature coupling and isotropic scaling technique after fifty PS recording intervals with an energy of 1.2 were completed. The goal of doing this was to provide precise temperature coupling.

### Simulation trajectory analysis

Schrodinger’s Maestro interface version 9.5 was utilized in order to render each and every image that was captured during the computational modelling simulation. The MD simulation was deemed to be of sufficient quality, and the Simulation Interaction Diagram (SID), which is a component of the Desmond module of the Schrodinger package, was utilized in order to conduct an analysis of the simulation event. According to this evaluation, the MD simulation is capable of meeting the requirements that are considered acceptable. On the basis of the trajectory output, the root mean square deviation (RMSD), root-mean-square fluctuation (RMSF), protein-ligand contacts (P-L contacts), and hydrogen-bond interactions were utilized in order to assess the stability of the complex structure. This investigation was conducted with the purpose of determining whether or not the intricate construction was capable of withstanding a variety of stresses without deteriorating in its shape.

### Root mean square deviation (RMSD) *analysis*

The RMSD is a statistic used in molecular dynamics (MD) modelling to calculate the average distance an atom moves in comparison to a standard over a given time period (Fukutani et al., 2021). Distance is relative when compared to a starting point in time. Following the alignment of the relative mean square deviation (RMSD) of the protein-fit ligand atoms from each time frame, a comparison to the reference time, in this case 100 nanoseconds is performed. This comparison is based on the RMSD of the protein’s structural atoms, which include the Ca, backbone, sidechain, and heavy atoms. It would be evident whether or not the alignment was satisfactory as soon as the reference time arrived. The RMSD required for an MD simulation of length x time steps can be determined using the equation shown below (Equation 9).

$${RMSD}_{x}=\sqrt{\frac{1}{N\;}}\sum_{i=1}^{N}\left(r_{i}^{\prime }\;\left(t_{x}\right)\right)-{\left.r_{i}\left(t_{ref}\right)\right)}^{2}$$ (9)

here, N specifies the total number of selected atoms, $t_{ref}$ denotes the reference time, and r' denotes the position of the selected ref atom in frame x. After superimposing the reference frame, Tx defines the recording intervals.

### Root mean square fluctuation *(RMSF* *) analysis*

In order to characterize and keep track of the local conformational shift that takes place within a protein structure, the RMSF is primarily applied (Ding and Peng, 2019). An MD simulation of a protein can be constructed by using the equation (Equation 10), which asks for the number of residues and the RMSF value.

$${RMSF}_{i}=\sqrt{\frac{1}{T\;}}\sum_{t=1}^{T}<\left(r_{i}^{\prime }\;\left(t\right)\right)-{\left.r_{i}\left(t_{ref}\right)\right)}^{2}>$$ (10)

In this case, T mainly denotes the trajectory time, r' denotes the chosen atoms’ position in the reference frame as overlaid on frame i, $t_{ref}$ denotes the reference or given time, and (<>) denotes the average of the square distance over residue b.

### Toxicity assessment

The amount of toxicity of a chemical substance can be measured by determining the extent to which it poses a risk to humans or animals, or by determining if it has the power to destroy an organ. Toxicity evaluation refers to both of these procedures. Prior to the initiation of a drug study, an investigation of the potential detrimental effects of chemical substances must be conducted. The conduct of a toxicity test is commonly recognized as one of the most important and critical components of the pharmaceutical production process. As a result, the web-based pkCSM server (Pires et al., 2015) was used to successfully complete the evaluation of the toxicity of the compounds chosen.

## Results and analysis

### Chemistry of extracted phytochemicals

**FIGURE 2:**
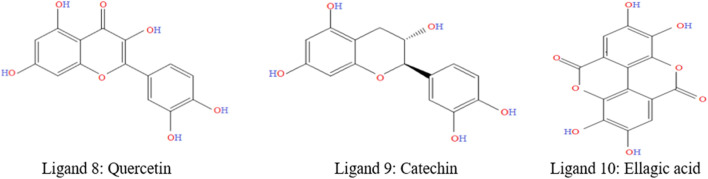
2D chemical structure of top selected ligands.

The diversity of natural phytochemicals, along with their intriguing biological roles, sets them apart from synthetic phytochemicals. It is difficult to draw clear functional and structure-activity relationships regarding the effects of phytochemicals on biological systems’ activity (Efferth and Koch, 2011). This is mostly due to the complex interactions that take place inside physiological systems as well as the high concentration of phytochemicals that have structural similarities. In addition, a significantly larger number of phytochemicals likely exist in nature, given the vast number already discovered. Technological advancements in synthesis, along with the development of more effective methods for isolation and analysis, have increased and help to identify the novel phytochemicals as lead compounds for the treatments of various diseases (Avidon et al., 1982). Figure 2 was generated with ChemDraw Ultra 12.0 and shows the chemical formulas and two-dimensional structures of top two ligands or phytochemicals and one FDA-approved medication (D1: Abemaciclib). The supplemental ST-2 contains an abundance of ligand-related information, and the 2D chemical structures of the selected ligands is depicted in Supplementary Figure S1.

### Geometry optimized structures of ligands

**FIGURE 3:**
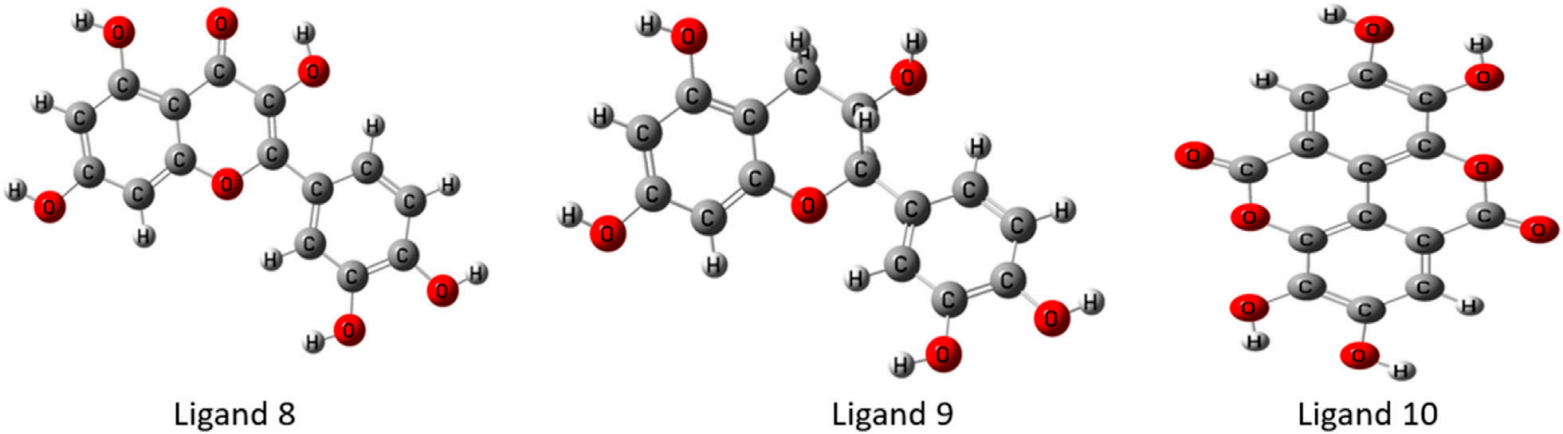
Geometry optimized chemical structures of top ligands.

In the discipline of computational chemistry, it is common practice to employ quantum mechanical methods to determine the thermodynamic, molecular orbital, and electrostatic properties of molecules. Each calculated derivative was strengthened and geometrically changed using the Gaussian 09 program to produce better results. Using DFT, we were able to boost the molecular orbital and thermal properties, allowing us to make predictions. This theory is compatible with both the hybrid model of B3LYP (Becke, 3-parameter, Lee-Yang-Parr) and the Gaussian version 09 polarization function basis set 6-311G (split-valence basis set) (Singh and Singh, 2017). Each chemical compound’s electrical energy, dipole moment, enthalpy, and free energy were calculated. Figure 3 depicts the optimal geometry and structure of the top most phytochemicals, and the all-optimized structure are shown in Supplementary Figure S2.

Optimized chemical structures of ligands are crucial in drug discovery, molecular docking, and computational chemistry, as they represent the most stable, energy-minimized conformations. By optimizing ligand structures, researchers ensure accurate interaction predictions with target biomolecules, enhancing binding affinity and specificity. This process helps in rational drug design, reducing experimental costs and time. Furthermore, optimized ligands improve the accuracy of pharmacokinetic and pharmacodynamic modeling, leading to more effective and safer therapeutic agents.

### Frontier molecular orbitals (FMOs) evaluations

**FIGURE 4:**
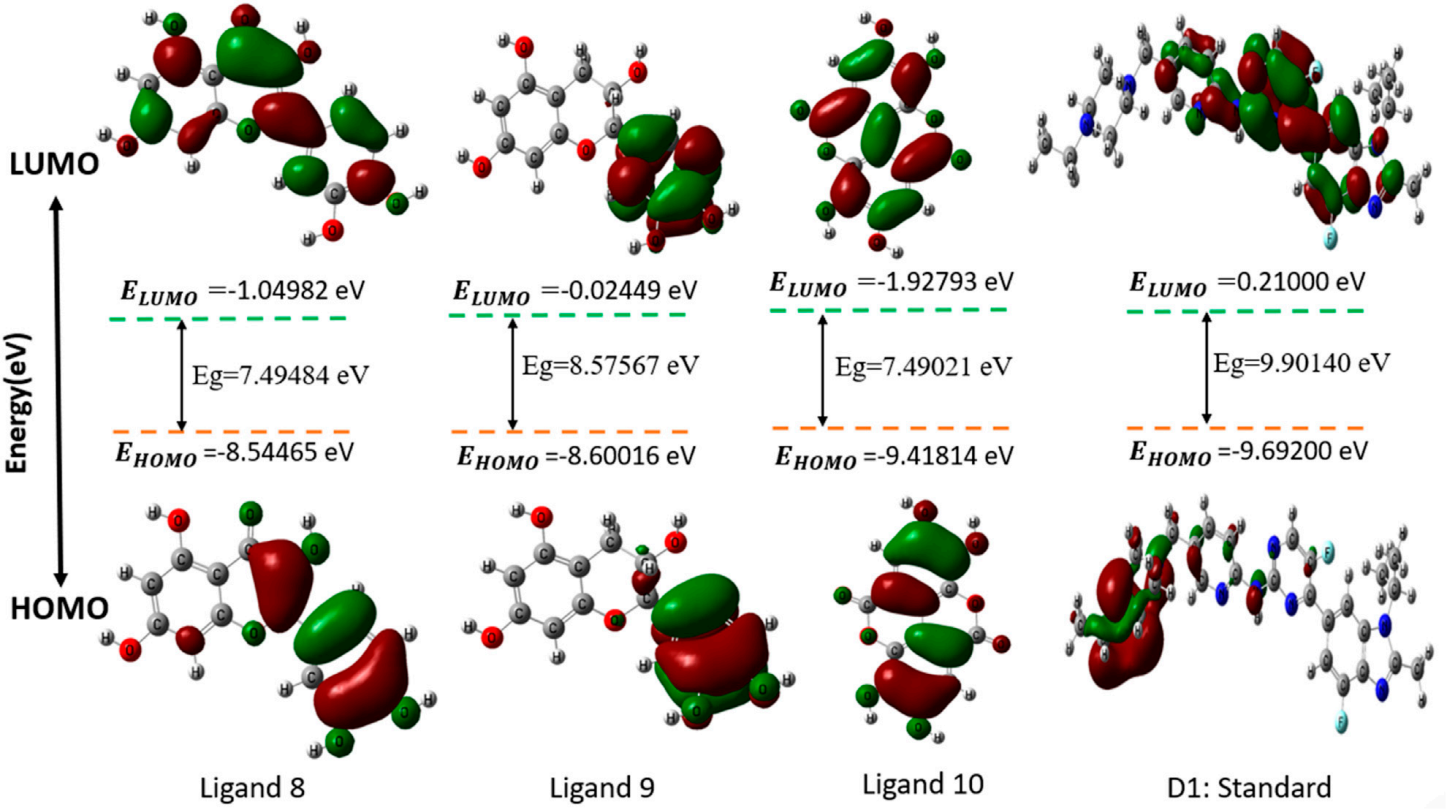
Diagram of HOMO-LUMO for selected compounds.

Chemical descriptors (HOMO and LUMO) and FMOs control the kinetic stability and chemical reactivity of molecules, respectively. Molecular orbitals that are least occupied are called LUMOs, and those that are most populated are called HOMOs. The electronic absorption of molecules releases one electron, which is caused by the HOMO state. The LUMO state accepts the electron simultaneously, and an energy gap forms as a result. These properties—kinetic stability, chemical reactivity, and atomic electrical transmission—are built upon this energy gap. The larger the energy difference, the more stable the molecule is when its HOMO and LUMO are far apart. The reason behind this is that the DFT method is used to calculate the energy gap. However, chemical stability is negatively affected by a small energy gap when the distance between a molecule’s HOMO and LUMO is small (Ahamed et al., 2023) (Kobir et al., 2023) (Yu et al., 2022). The small energy gap is the root cause of the chemical instability. In Figure 4, the color radish brown is used to represent the molecules’ positive node in both HOMO and LUMO situations, whereas the color deep green is used to represent the molecules’ negative node.

### Quantum mechanics (QM) and chemical reactivity analysis

The table below covers several medications and phytochemicals, as well as information regarding their reactivity and frontier molecular orbitals (HOMO-LUMO). The sign A denotes HOMO energy, the letter I denotes LUMO energy, the letter μ represents chemical potential, the letter η denotes hardness, the letter σ denotes softness, the letter X denotes electronegativity, and the letter ω denotes electrophilicity. When we examine the energy gap between each of the fifteen molecules, we can find that Ligand No. 07 has the highest chemical stability at 11.3746 eV and the lowest at 7.49021 eV. This information can be found in Table 2. The Supplemental ST-3 has an extremely comprehensive computation.

**TABLE 2:** Frontier molecular orbitals and reactivity descriptor analysis.

Ligand no.	A = HOMO (eV)	I = LUMO(eV)	E gap = (I-A)eV	Chemical potential (μ) = - (I + A)/2	Hardness (η) = (I-A)/2	Softness (σ) = 1/μ	Electronegativity (Χ) = (I + A)/2	Electrophilicity (ω) = μ2/2η
1	−9.14657	−1.08410	8.06247	5.11534	4.03123	0.19549	−5.11534	3.24549
2	−10.4680	−1.08927	9.37868	5.77861	4.68934	0.17305	−5.77861	3.56045
3	−9.20290	−0.93226	8.27063	5.06758	4.13532	0.19733	−5.06758	3.10501
4	−9.76780	0.378240	10.1460	4.69478	5.07302	0.21300	−4.69478	2.17237
5	−8.92997	−0.85934	8.07063	4.89465	4.03531	0.20430	−4.89465	2.96849
6	−9.05350	−0.93036	8.12315	4.99193	4.06157	0.20032	−4.99193	3.06770
7	−10.4799	0.89471	11.3746	4.79261	5.68732	0.20865	−4.79261	2.01933
8	−8.54465	−1.04982	7.49484	4.79723	3.74742	0.20845	−4.79723	3.07057
9	−8.60016	−0.02449	8.57567	4.31233	4.28784	0.23189	−4.31233	2.16848
10	−9.41814	−1.92793	7.49021	5.67303	3.74510	0.17627	−5.67303	4.29672
11	−8.86466	−1.11757	7.74709	4.99111	3.87354	0.20036	−4.99111	3.21556
12	−9.22358	−1.27132	7.95226	5.24745	3.97613	0.19057	−5.24745	3.46263
13	−9.14793	−1.18669	7.96124	5.16731	3.98062	0.19352	−5.16731	3.35388
14	−8.88371	−1.14995	7.73375	5.01683	3.86688	0.19933	−5.01683	3.25438
15	−9.27120	−1.36084	7.91035	5.31602	3.95518	0.18811	−5.31602	3.57254
D-1	−9.69200	0.21000	9.90140	4.74100	4.95100	0.21100	−4.74100	2.27000

The HOMO energy varies between −10.4799 eV (Ligand 7) and −8.54465 eV (Ligand 8), while the LUMO energy ranges from 0.89471 eV (Ligand 7) to −1.92793 eV (Ligand 10). The energy gap (E_gap) spans from 7.49021 eV (Ligand 10) to 11.3746 eV (Ligand 7), indicating significant variations in electronic stability and reactivity among the ligands. The chemical potential (μ) ranges from −5.77861 eV (Ligand 2) to −4.31233 eV (Ligand 9), with electronegativity (Χ) following the same trend. Hardness (η) varies from 3.74510 eV (Ligand 10) to 5.68732 eV (Ligand 7), while softness (σ) exhibits an inverse trend, emphasizing the ligands’ adaptability to electronic perturbations. Electrophilicity index (ω) values range from 2.01933 eV (Ligand 7) to 4.29672 eV (Ligand 10), indicating differences in their propensity to accept electrons. Notably, D-1 exhibits moderate values across all parameters, serving as a reference for comparative evaluation. These quantum chemical descriptors provide insights into the ligands’ stability, reactivity, and potential applications in coordination chemistry, catalysis, and molecular design.

### Analysis of molecular electrostatic potential (MEP)

**FIGURE 5:**
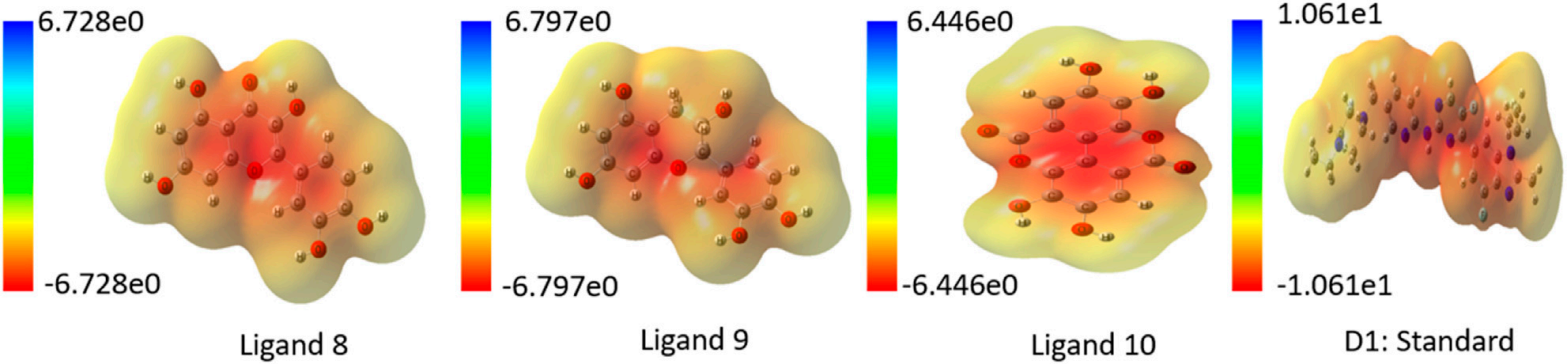
Charge distribution in Molecular Electrostatic Potential (MEP) for top ligands.

An essential part of computer-aided drug design is determining the ligands’ molecular electrostatic potential (MEP), which yields a charge distribution map based on electron availability and scarcity. Additionally, it shows where the protein and ligand bind and how the charges are distributed in three-dimensional ligand structures. When applied to ligand surface analysis, MEP analysis can further help pinpoint where ligands are vulnerable to attack from electrophiles and nucleophiles (Lakshminarayanan et al., 2021; Guerrab et al., 2022). Utilizing quantum chemistry techniques, the MEP map was generated, and Gaussian functions were assessed with the use of Gaussian fundamental sets. In this study, blue represents positive charge, red negative charge, and green neutral charge. A lower negative charge than a larger positive charge is observed in all of the discovered ligands, as shown in the MEP map of top ligands in Figure 5. In Supplementary Figure S3 contains MEP map for all ligands.

### Analysis of PASS prediction data

The PASS prediction assessment displays the details of the antiviral, antibacterial, antifungal, antiparasitic, anticarcinogenic, anticancer (breast cancer), and inhibitory effects of drugs and ligands on breast cancer-resistant proteins. It is used to assess the potential biological activity against the targeted disease. According to our investigation, the Ligand No. 07 has low to moderate antiviral activity (Pa > 0.454), Ligand No. 11 and 14 have moderate to high antibacterial activity (Pa> 0.599), and Ligand No. 15 has low to significantly high antifungal activity (Pa > 0.678). To top it all off, most ligands have excellent anti-carcinogenic activity, meaning they won't cause cancer when taken orally. Ligands 05 (Pa > 0.649), 08 (Pa > 0.577), 11, and 14 (Pa > 0.502), as well as No (Pa> 0.526), exhibit strong anticancer properties, according to additional research. After reviewing the PASS prediction data for breast cancer-resistant protein has been selected, and we found that Ligand No. 05 has a very good Pa value (Pa > 0.516) as illustrate in Supplementary Table S4.

### Evaluation of ligands’ drug likeliness and pharmacokinetics properties

Pharmacokinetics (PK), an important parameter in medicine design, explains the time it takes for the body to absorb, distribute, metabolize, excrete, and contaminate a drug or foreign chemical after administration. This word is commonly used by pharmacists. As a result, PK promotes effective drug design (Lavé et al., 2016). Table 4 displays information for certain ligands and the medicine on drug likelihood, pharmacokinetics, Lipinski’s rule of five, and other topics. A ligand must meet ADME standards before it may be evaluated for drug candidate certification. The five Lipinski rules must be followed: a molecular weight ranging from 150 to 500 g/mol, a limited number of hydrogen bond donors, a number of rotatable bonds, a high bioavailability score, and a topological surface area value ranging from 20 Å^2^ to 130 Å^2^ (Singh et al., 2022; Al Hasib et al., 2022).

The Table 3 reveals that, with the exception of Ligands 11, 12, 13, 14, and 15, all of the ligands follow Lipinski’s rule of five. In terms of molecular weight, all of the ligands have the necessary molecular weights (150–500 g/mol), with the exception of drug D-1 (506.59 g/mol) and Ligand No. 15 (594.52 g/mol). As a result, with the exception of Ligands 04, 11, 14, and 15 (which have six rotatable bonds) and Ligand D-1 (17 rotatable bonds), no ligand can have more than three rotatable bonds. Except for Ligands 11 (12) and 12, the hydrogen bond acceptor can only receive a maximum of 10 hydrogen bonds from any of the ligands. Ligands are the numbers 12, 15, and N0 13. Except for ligands 7, 8, 9, 11, 12, 13, 14, and 15, no other ligand contributes more than five hydrogen bonds. Ligands 8, 10, 11, 12, 13, 14, and 15 do not match the drug development criteria since their topological surface areas exceed the range of 202 Å^2^ to 130 Å^2^. Apart from Ligands 11, 12, 13, 14, and 15, all of the other ligands had acceptable bioavailability values. After evaluating the facts offered previously, a conclusion can be reached. Several ligands have been removed from consideration as prospective pharmaceutical candidates; nevertheless, ligands 01, 02, 03, 05, and 06 remain on the list.

**TABLE 3:** Data of ligands’ drug likeliness, pharmacokinetics properties, and Lipinski’s rule.

Ligand no.	Molecular weight (g/mol)	Number of rotatable bonds	Hydrogen bond acceptor	Hydrogen bond donor	Topological polar surface area (Å^2^)	Lipinski’s rule		Bioavailability score
						Results	Violation	
01	170.12	1	5	4	97.99	Yes	00	0.56
02	174.15	1	5	4	97.99	Yes	00	0.56
03	154.12	1	4	3	77.76	Yes	00	0.56
04	213.23	4	5	3	86.63	Yes	00	0.56
05	260.20	0	6	4	111.13	Yes	00	0.55
06	184.15	2	5	3	86.99	Yes	00	0.55
07	180.16	1	6	5	110.38	Yes	00	0.55
08	302.24	1	7	5	131.36	Yes	00	0.55
09	290.27	1	6	5	110.38	Yes	00	0.55
10	302.19	0	8	4	141.34	Yes	00	0.55
11	464.38	4	12	8	210.51	No	02	0.17
12	422.34	2	11	8	201.28	No	02	0.17
13	436.37	3	11	7	190.28	No	02	0.17
14	464.38	4	12	8	210.51	No	02	0.17
15	594.52	6	15	9	249.20	No	03	0.17
D-1	235.07	4	8	6	180.93	Yes	1	0.55

### Molecular docking analysis of selected proteins and ligands

To identify possible breast cancer medication candidates, auto-docking was carried out with selected proteins susceptible to breast cancer (PDB ID: 3W32). Table 4 displays the molecular docking simulation data with binding affinities. In molecular docking, three chosen proteins have the highest binding affinities when interacting with Ligands Nos. 10, 11, and 12. However, Ligands 11 and 12 do not match the ADME criteria listed in Table 4. Ligands No. 08, 09, and 10 meet all drug property criteria in this study, having binding affinities of −8.5 kcal/mol, −8.4 kcal/mol, and −8.8 kcal/mol, respectively.

**TABLE 4:** Data of binding energy and name of interacted ligand for breast cancer protease (3W32).

Ligand No.	Binding affinity (kcal/mol)	No. of H bond	No. of hydrophobic bond	No. of van der waal bond	Total bonds
1	−6.1	05	02	Absent	07
2	−6.4	03	00	Absent	03
3	−6.3	03	04	Absent	07
4	−5.8	02	05	Absent	07
5	−7.6	02	05	Absent	07
6	−5.9	03	03	Absent	06
7	−5.7	03	00	Absent	03
8	−8.5	02	08	Absent	10
9	−8.4	04	05	Absent	09
10	−8.8	01	10	Absent	11
11	−8.6	01	03	Absent	04
12	−8.7	02	07	Absent	09
13	−8.5	04	04	Absent	08
14	−6.1	04	03	Absent	07
15	−6.4	04	08	Absent	12
D-1	−7.8	04	00	Absent	04

### Protein-ligand interactions diagram

**FIGURE 6:**
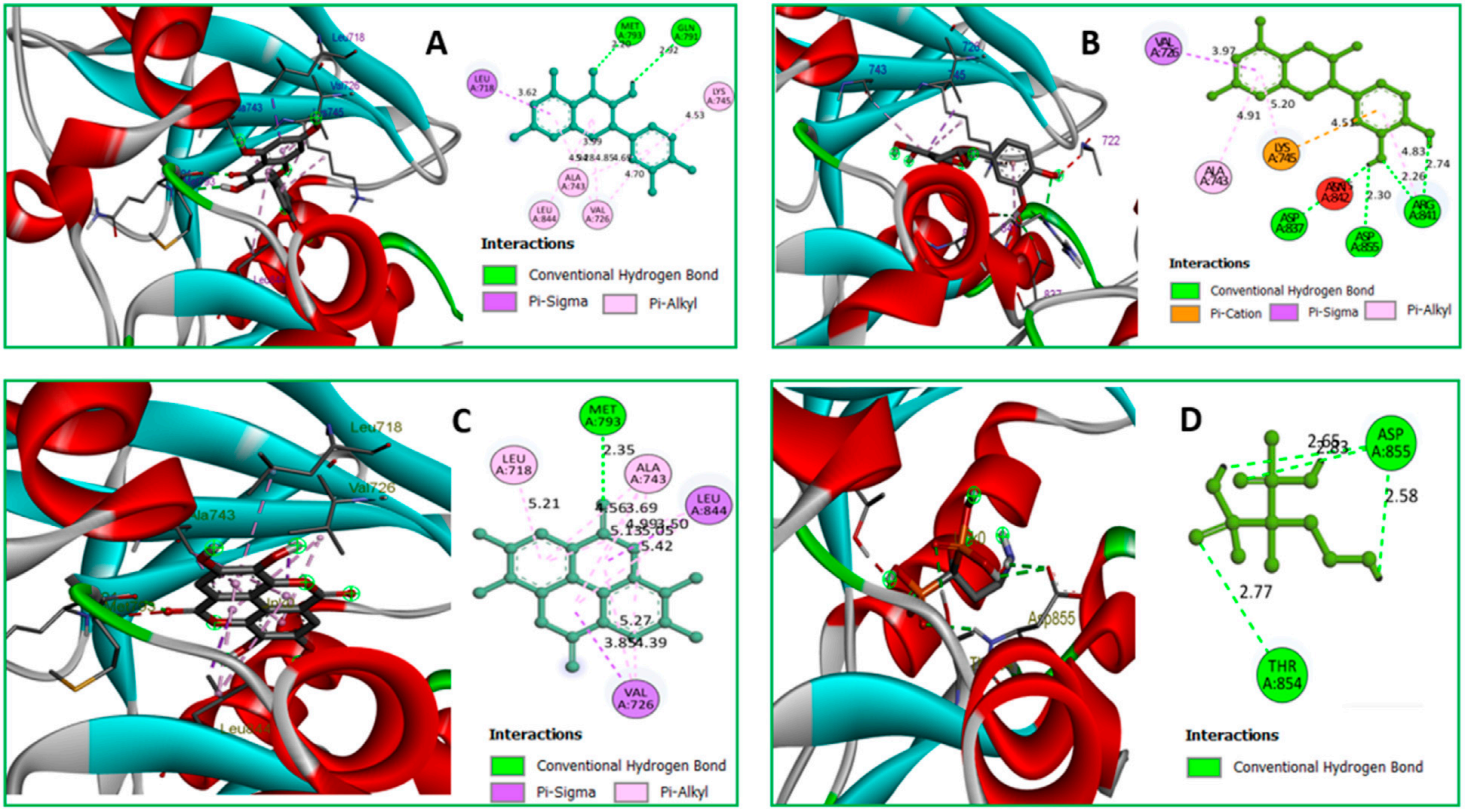
**(A)** The interaction between the 3w32 protein and Ligand 08 compounds. The 3D interaction has represented left side of the figure, where 2D interaction has depicted in right side of the figure accordingly. **(B)** The interaction between the3w32 protein and Ligand 09 compounds. The 3D interaction has represented left side of the figure, where 2D interaction has depicted in right side of the figure accordingly. **(C)** The interaction between the 3w32 protein and Ligand 10 compounds. The 3D interaction has represented left side of the figure, where 2D interaction has been depicted in the right side of the figure accordingly, and **(D)** The interaction between the 3w32 protein and Standard D1 compounds. The 3D interaction has represented left side of the figure, where 2D interaction has been depicted in the right side of the figure accordingly.

Protein-ligand interactions (PLIs) and protein-protein interactions (PPIs) play critical roles in identifying possible drug candidates for a target protein in structure-based drug design and drug discovery (Zhao and Bourne, 2022; Fu et al., 2018). This is why it is referred to as a critical component of the process. On the other hand, this part of the therapeutic goal is very important in and of itself. Because of the unique structural properties of protein interactions with ligands, it is today regarded as one of the most difficult areas of drug development. Bond distance research on the principal protease of breast cancer proteins have been conducted, with the primary focus of the studies being on the interaction of medicinal medicines with 3w32. Figure 6 depicts the key interactions that proteins and ligands have with amino acid residues.

### Interacted amino acids with bond distance

Data on hydrogen bonds, hydrophobic bonds, and bond distances between amino acids are shown in Table 5. One of the most important factors in selecting a potential drug candidate is the bond distance, with a value between 3.1 Å to 3.55 Å indicating a weak link and a value between 2.5 Å to 3.1 Å indicating a strong binding, according to previous studies (da Cunha Xavier et al., 2024). Since the ADME screening and molecular docking did not include any other Ligands, we looked at how proteins with PDB ID 3W32 interacted with Ligands No. 01, to 15, and standard D1. From the, it shows that ligand 08 forms GLN791 (2.92 Å) and MET793 (2.20 Å) two strong hydrogen bonds, LEU718 (3.62 Å), VAL726 (4.70Å), VAL726 (3.99 Å), VAL726 (4.95 Å), LEU844 (4.69Å), ALA743 (4.28 Å), ALA743 (4.85 Å), LYS745 (4.53 Å) other bonds with protein 3W32. The ligand 09 forms ASP837 (2.55 Å), ASP855 (3.30 Å), AR841 (2.26 Å), and ARG841 (2.74 Å) strong bonds, and VAL726 (3.97 Å), ARG841 (4.83 Å), ALA743 (4.91 Å), LYS745 (5.20 Å), and LYS745 (4.51 Å) other bonds. On the other hand, ligand 10 forms MET793 (2.35Å) strong bond, and LEU718 (5.21Å), VAL726 (5.27Å), VAL726 (4.39Å), ALA743 (4.56Å), ALA743 (5.13Å), ALA743 (3.69 Å), ALA743 (4.99 Å), LEU844 (5.05 Å), VAL726 (3.85 Å), and LEU844 (3.50 Å) other bonds. Besides the standard D1 form ASP855 (2.65 Å), ASP855 (2.83 Å), ASP855 (2.58 Å), and THR854 (2.77 Å) strong bond with protein. For more details on the various bond classifications and types, see supplementary SF-4 and Supplementary Table S5.

**TABLE 5:** Protein-ligand interactions and interacting bonds.

Protein PDB ID: 3W32									
No.	Hydrogen bond		Hydrophobic bond		No.	Hydrogen bond		Hydrophobic bond	
	Interacting residue of Amino acid	Distance A°	Interacting residue of Amino acid	Distance A°		Interacting residue of Amino acid	Distance A°	Interacting residue of Amino acid	Distance A°
01	MET 766 THR 790 THR 854 ASP 855 PHE 856	2.94 3.00 2.57 3.24 1.99	MET 766 LEU 777	5.17 4.66	09	ASP 837 ASP 855 ARG 841 ARG 841	2.55 3.30 2.26 2.74	VAL 726 ARG 841 ALA 743 LYS 745 LYS 745	3.97 4.83 4.91 5.20 4.51
02	MET 766 LEU 777 THR 790	2.73 2.65 2.70	Absent		10	MET 793	2.35	LEU 718 VAL 726 VAL 726 ALA 743 ALA 743 ALA 743 ALA 743 LEU 844 VAL 726 LEU 844	5.21 5.27 4.39 4.56 5.13 3.69 4.99 5.05 3.85 3.50
03	MET 766 LEU 777 ASP 855	2.79 2.51 2.64	MET 766 LEU 777 LEU 788 PHE 856	5.08 3.62 5.47 5.48	11	ASN 842	2.88	VAL 726 ALA 743 LYS 745	4.33 4.79 4.63
04	MET 793 MET 793	1.93 1.94	VAL726 ALA743 ALA 743 LEU 844 LYS745	4.05 3.79 4.43 4.94 4.53	12	LYS 745 ASP 855	2.49 3.65	VAL 726 ALA 743 ALA 743 LYS 745 LYS 745 LEU 844 VAL 726	4.44 4.73 4.90 5.30 4.59 5.24 3.83
05	MET 793 ASP 855	2.22 2.59	LEU 718 VAL 726 VAL 726 LEU 844 LEU 844	5.42 4.55 4.91 4.69 5.73	13	LYS 745 ASP 800 ARG 841 ASN 842	4.11 3.01 2.49 3.54	VAL 726 LYS 845 LYS 845 VAL 726	4.77 4.80 4.74 3.88
06	MET 766 ASP 855 ASP 855	2.73 3.51 3.19	LEU 777 MET 766 PHE 856	4.75 5.29 5.33	14	MET 793 ASN 842 ASP 855 GLY 721	2.70 2.69 2.20 3.23	VAL 726 ALA 743 LYS 745	4.31 4.80 4.62
07	ASN 771 VAL 774 LYS 852	2.11 2.21 2.08	Absent		15	LEU 718 LYS 745 ASP 800 ASP 855	2.93 2.69 2.14 2.47	VAL 726 VAL 726 ALA 743 ALA 743 LEU 844 LEU 844 ARG 841 LYS 745	4.63 4.60 5.35 5.25 5.00 4.93 4.11 4.95
08	GLN 791 MET 793	2.92 2.20	LEU 718 VAL726 VAL726 VAL726 LEU 844 ALA743 ALA 743 LYS745	3.62 4.70 3.99 4.95 4.69 4.28 4.85 4.53	D-1	ASP855 ASP 855 ASP 855 THR854	2.65 2.83 2.58 2.77	Absent	

### Molecular dynamic simulation

MD simulation modelling, which is employed in computer-aided drug discovery, may study the protein-ligand complex’s stability and intermolecular interactions in real time. In a controlled situation, it can also detect conformational changes in complicated systems. A 100 ns molecular dynamics simulation was used to investigate the protein’s structural changes during its interaction with the chosen ligand. Multimolecular activity was measured on final images acquired from the appropriate 100 ns trajectories. It was examined the findings of a molecular dynamics (MD) simulation, and this comprised solvent accessible surface area (SASA), radius of gyration (Rg), root mean square fluctuation (RMSF), and root mean square deviation.

### Analysis of protein’s RMSD

**FIGURE 7:**
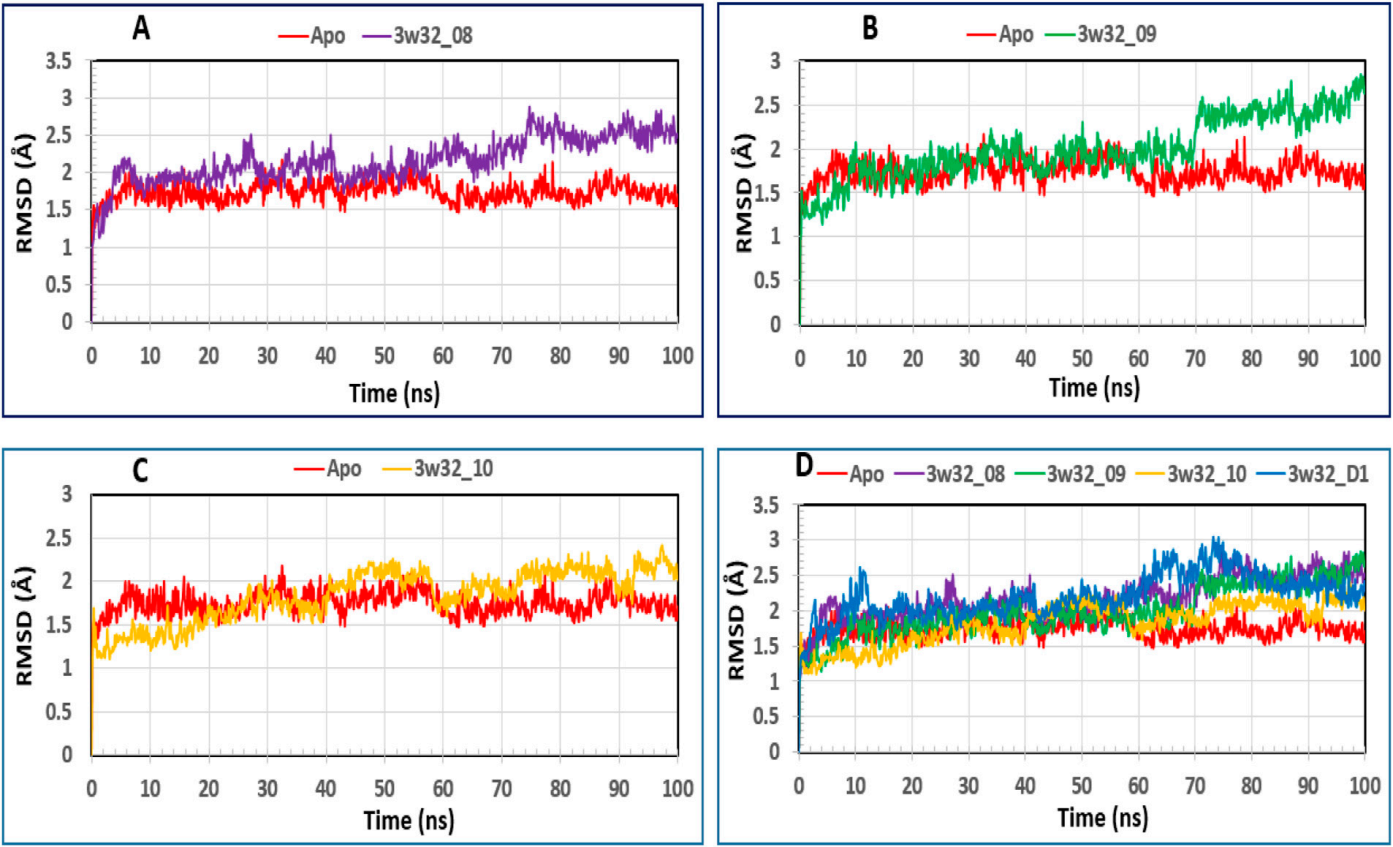
The RMSD measurements of the Cα atoms in the four compounds selected to form a complex with the breast cancer protein are illustrated: Herein, showing the RMSD of breast cancer protein 3w32 as Apo (Red) in complex with the compounds **(A)** 3w32_08 (purple), **(B)** 3w32_09 (Green), and **(C)** 3w32_10 (Orange), where **(D)** 3w32_D1 standard (Blue) representing all the compounds and protein RMSD together.

The average dislocation change of a chosen group of atoms over a certain period of time in relation to a reference time can be found using an RMSD value. A protein-ligand complex’s optimal RMSD shift falls between 1 Å and 3 Å, or 0.1 and 0.3 ns. When the RMSD value exceeds the permitted limit, the protein’s structure has undergone a significant alteration. To determine the RMSD value of the essential protein in association with the selected molecule and ligands 08, 09, and 10, we performed a 100 ns MD simulation. Figure 7 displays the RMSDs, or relative standard deviations, of several complexes. The following protein-ligand complexes are displayed: Apo-protein (red), protein-3w32 and ligand-08 (purple), protein-3w32 and ligand-10 (green), and protein-3w32 and ligand-D1 (standard) (blue). In Figure 7D, you can see all of these complexes unified. Prior to making comparisons, the red Apo protein Apo’s RMSD is shown. Figure 7A shows that the average RMSD value ranges from 1.5 Å to 2.6 Å when comparing the protein-ligand combination 3w32_08 (purple color) to Apo (red color). Figure 7B shows that between 1.7 Å and 2.8 Å, the protein-ligand combination 3w32_09 (green) compared to Apo (red). Comparing the protein-ligand combination 3w32_10 (orange color) with Apo (red color), Figure 7C shows the RMSD variation within 1.4 Å to 2.3 Å. The four compounds (3w32_D1 in blue, Apo in red, 3w33_08 in purple, 3w32_09 in green, and 3w32_10 in orange) had an average RMSD value between 1.5 Å to 2.6 Å. This means that the compound’s value fluctuation falls within the target range, as stated in references (Timofeev et al., 2023; Akash et al., 2023).

### Ligand RMSD analysis

**FIGURE 8:**
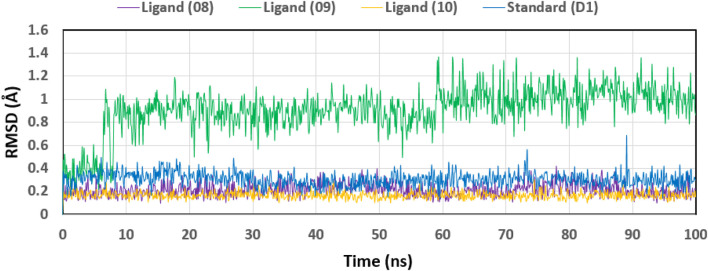
Displaying the RMSD values of the four compounds that were chosen and isolated from the complex system’s Ca atoms. The four compounds—Lagend Nos. 08, 09, 10, and D1—were shown as purple, green, orange, and blue, respectively.

We analyzed the tested drugs and controls with respect to their RMSDs to find out which one was more stable. See Figure 8 for the RMSD values for Ligand No. 08 (purple), Ligand No. 09 (green), Ligand No. 10 (orange), and conventional D1 (blue). The compounds’ RMSD was determined after aligning the docking complex with the Apo standard protein backbone. To find the optimal RMSD for each chemical, complex observation was utilized in this situation. The green RMSD of Ligand No. 09 fluctuates, although it stays within the permissible range of 0.5–1.4 Å (Liu et al., 2017; Sherman et al., 2006). Because of minute differences, Ligands No. 08 (purple) and No. 10 (orange) are rather inflexible. There isn't a whole lot of movement in the blue control D1 here. After looking at the RMSD values, this study revealed that all of the ligands were stable, but that Ligand No. 09 (green) was the most stable.

### Protein Cα RMSF analysis

**FIGURE 9:**
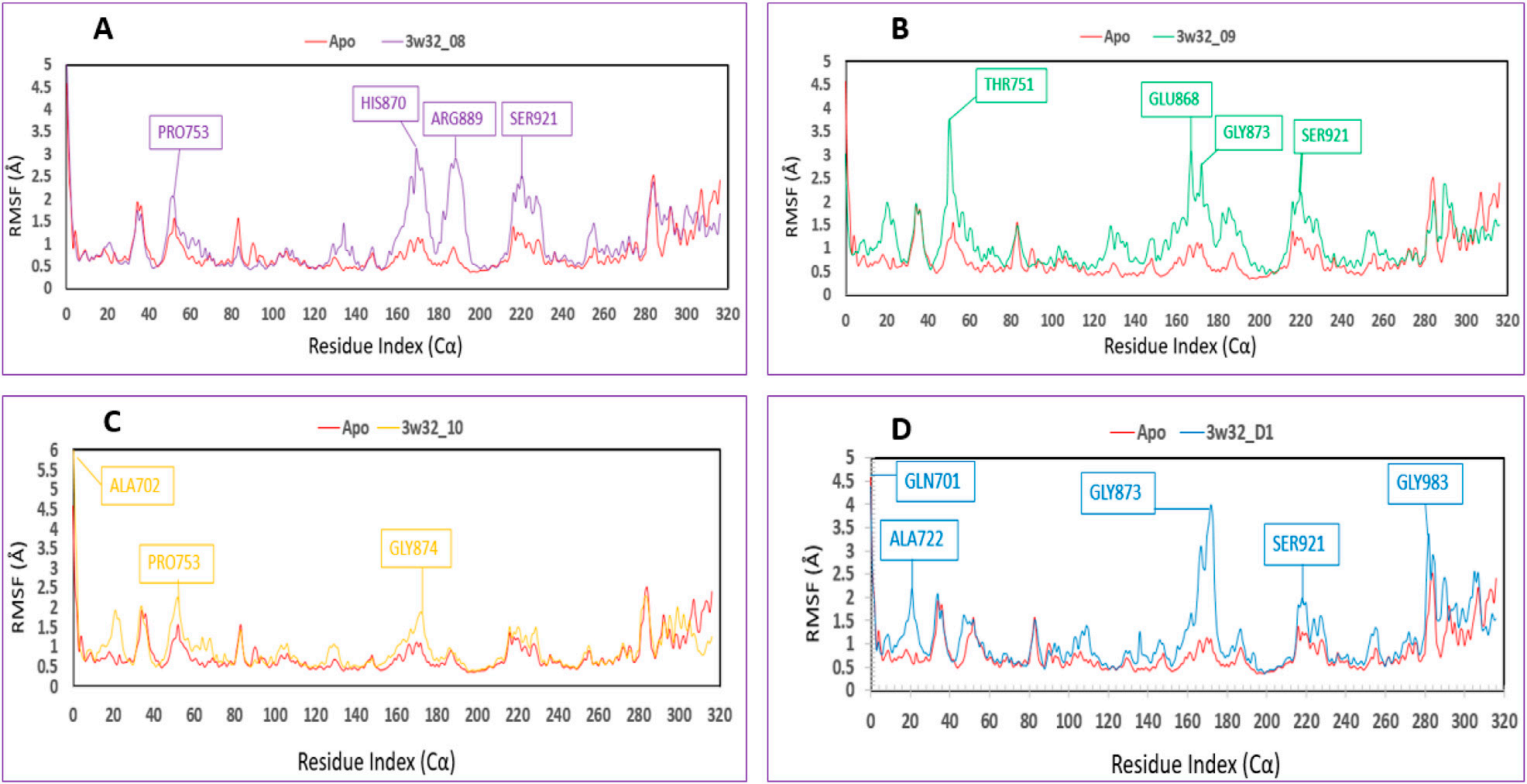
Showing the RMSF values that were taken out of the Cα atoms of the four compounds and were chosen to be in complex with the 3w32 protein. Herein, comparison of the RMSF of 3w32 Apo (Red) in complex with the compounds **(A)** 3w32_08 (purple), **(B)** 3w32_09 (Green), and **(C)** 3w32_10 (Orange), where **(D)** 3w32_D1 standard (Blue).

The root mean square fluctuation, or RMSF, can be used to identify and define the local modifications that occur along the protein chain when drugs interact with certain residues. So, to see how the shape of proteins changes when certain ligands attach to a specific remaining site, we calculated and displayed the RMSF value of the initial protein-ligand complexes in Figure 9. The variations of different chemical complexes with the targeted protein 3w32 are compared in this study. Figure 9A compares the 3w32_08 (purple) complex protein to the Apo (red), revealing that only PRO753, HIS870, ARG889, and SER921 were more fluctuating. That is, 98.73% of the residues were stable. Figure 9B compares 3w32_09 (green) complex protein to Apo (red) protein and reveals that THR751, GLU868, GLY873, and SER921 residues are more volatile, implying that 98.73% of residues are stable. Figure 9C compares 3w32_10 (orange) complex proteins to Apo (red), revealing that ALA702, PRO753, and GLY874 residues were more variable, indicating that 99.05% of the residues were stable. The conventional 3w32_D1 complex was compared to Apo (red) protein in Figure 9D, and it was discovered that GLN701, ALA722, GLY873, SER921, and GLY983 residues were more fluctuating, implying that 98.41% of the residues were stable. Protein stiffness is indicated by the substantially smaller variability of residues in the complex structure compared to the native structural components (Apo). Because the N- and C-terminal domains are located at the beginning and end of the protein, the majority of the changes are found there. As a result, ligands 08, 09, and 10 are suitable candidates for a molecule in which the possibility of a specific atom being displaced in a real-world environment is low.

### Protein-ligand contacts evaluations

**FIGURE 10:**
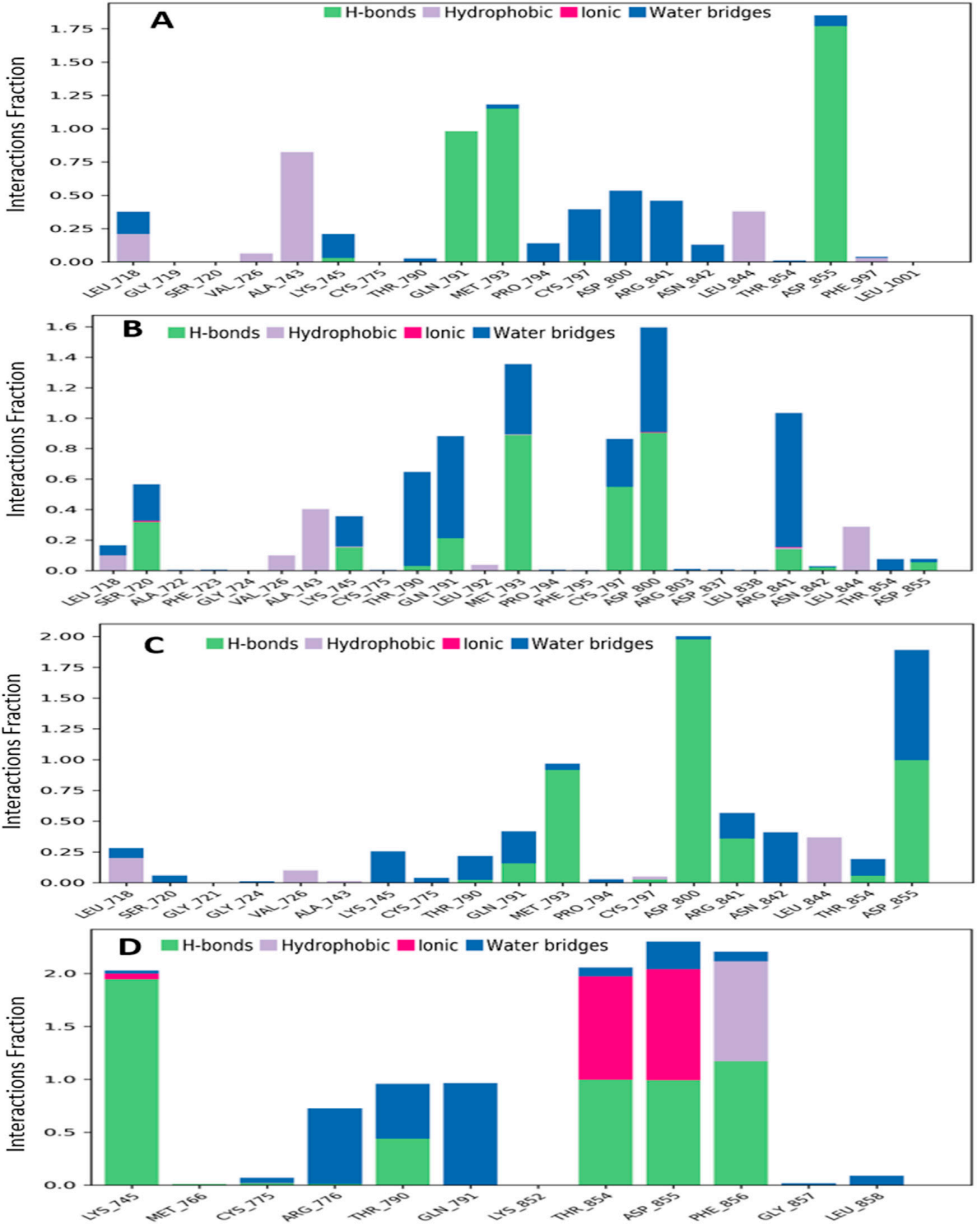
Graphs exhibiting the information about the protein–ligand interaction by 100 ns time of MD simulation. Herein, representing the compounds **(A)** ligand 08, **(B)** ligand 09, **(C)** ligand 10 and **(D)** standard D1 with the interactions of protein ID 3w32.

The interactions between the four selected ligands (Ligand 08, Ligand 09, Ligand 10, and Standard D1) and the breast cancer proteins 3w32 were monitored throughout the SID. Drug selectivity, metabolization, and adsorption seem to be significantly influenced by hydrogen-bonding properties in drug design, as demonstrated by the MD simulation’s identification of hydrogen bonds, hydrophobic, ionic, and water bridge interactions. The simulation amply demonstrated the hydrogen bonding connection stated for both compounds up to the very last AA residue. In all structures, the protein residue and the ligand form a variety of interactions, including as hydrogen bonds, hydrophobic interactions, ionic interactions, and water bridges, as Figure 10 shows. The complex 3w32_08 for ligand 08 produced multiple (more than two) interactions at the residues of ASP855, MET793, GLN790, ALA743, ASP800, ASG841, CYS797, LEU844, LEU718, LYS745, PRO794, and ASN842 with an interaction fraction (IF) value of 1.85, 1.25, 1.00, 0.85, 0.55, 0.50, 0.48, 0.45, 0.25, 0.20, and 0.20, respectively. This means that for 155%, 125%, 100%, 85%, 55%, 50%, 48%, 48%, 45%, 20%, and 20% of the simulation time, the specific interaction is maintained by the multiple contacts of the same subtype with the ligand as indicated in Figure 10A. Multiple interactions of the 3w32_09 complex have been observed in the case of ligand 09 at the positions of ASP800 (1.6), MET793 (1.4), ARG841 (1.0), GLN791 (0.98), CYS797 (0.98), THR790 (0.7), SER720 (0.6), ALA743 (0.5), LYS745 (0.45), LEU844 (0.4), LEU718 (0.19), VAL726 (0.13), THR854 (0.10), and ASP855 (0.10) residues maintained by 160%, 140%, 100%, 98%, 98%, 70%, 60%, 50%, 45%, 40%, 19%, 13%, 10%, and 10% of the simulation time the in particular interaction indicated in Figure 10B. For ligand 10, it has been found that multiple interactions of the 3w32_10 complex are maintained by 200%, 198%, 100%, 60%, 50%, 50%, 45%, 30%, 30%, 25%, 15%, and 10% of the simulation time in the specific interaction shown in Figure 10C. These interactions are at the positions of ASP800 (2.00), ASP855 (1.98), MET793 (1.00), ARG841 (0.60), GLN791 (0.50), ASN842 (0.50), LEU844 (0.45), LEU718 (0.30), LYS745 (0.30), THR790 (0.25), THR854 (0.25), and SER720 (0.10) residues. Additionally, it has been discovered that, in the case of the standard D1, multiple interactions of the 3w32_D1 complex are maintained by 285%, 250%, 230%, 210%, 100%, 70%, 100%, and 5% of the simulation time in the specific interaction shown in Figure 10D. These interactions occur at the positions of ASP855 (2.85), PHE856 (2.50), THR854 (2.3), LYS745 (2.1), THR790 (1.00), GLN791 (1.00), ARG776 (0.70), LEU858 (0.10), and CYS775 (0.05) residues.

### Ligand properties analysis

We assessed the stability of the four compounds (ligand 08, ligand 09, ligand 10, and standard D1) in the MD simulation using ligand characteristics. We employed the Radius of Gyration (rGyr), Intramolecular Hydrogen Bonds (intraHB), Molecular Surface Area (MolSA), Solvent Accessible Surface Area (SASA), and Polar Surface Area (PSA) to examine the characteristics of the ligands. In this analysis the molar surface area (MolSA) as depicted in SF-5 and the Polar Surface Area (PSA) as shown in SF-6, were used to analyze the ligand properties, all of which were found to be favorable for the ligands.

### Radius of gyration (Rg)

**FIGURE 11:**
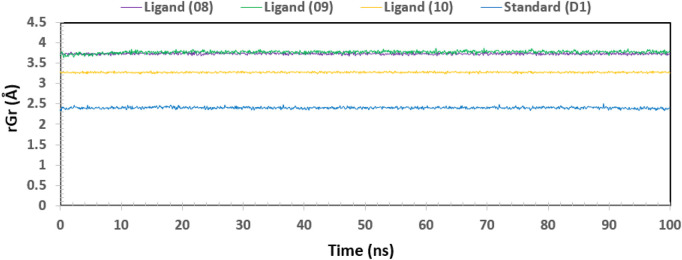
The radius of gyration (Rg) of the protein–ligand complex was calculated from the 100 ns simulation. The Rg value of the selected four compounds ligand (08), ligand (09) ligand (10) and standard (D1) in complex with the protein ID 3w32 represented by a purple, green, orange, and blue color, respectively.

One approach to think about the radius of gyration (Rg) of a protein-ligand complex system is in terms of atomic distribution along its axis. Rg is a useful tool and an important measure of the structural function of a macromolecule that can be used to predict changes in complex stiffness. Thus, we also investigated the stability of four ligands in their interaction with the target protein via Rg throughout the 100 ns simulation time depicted in Figure 11: ligand No. 08 (purple color), ligand No. 09 (green color), ligand No. 10 (orange color), and standard D1 (blue color). The compounds Ligand No. 08, Ligand No. 09, Ligand No. 10, and standard D1 were found to have average Rg values of 3.75, 3.76, 3.25, and 2.4, respectively. This implies that, upon binding the chosen compounds, the protein’s active site did not experience any appreciable conformational changes (Mendichi et al., 2003).

### Solvent accessible surface area (SASA)

**FIGURE 12:**
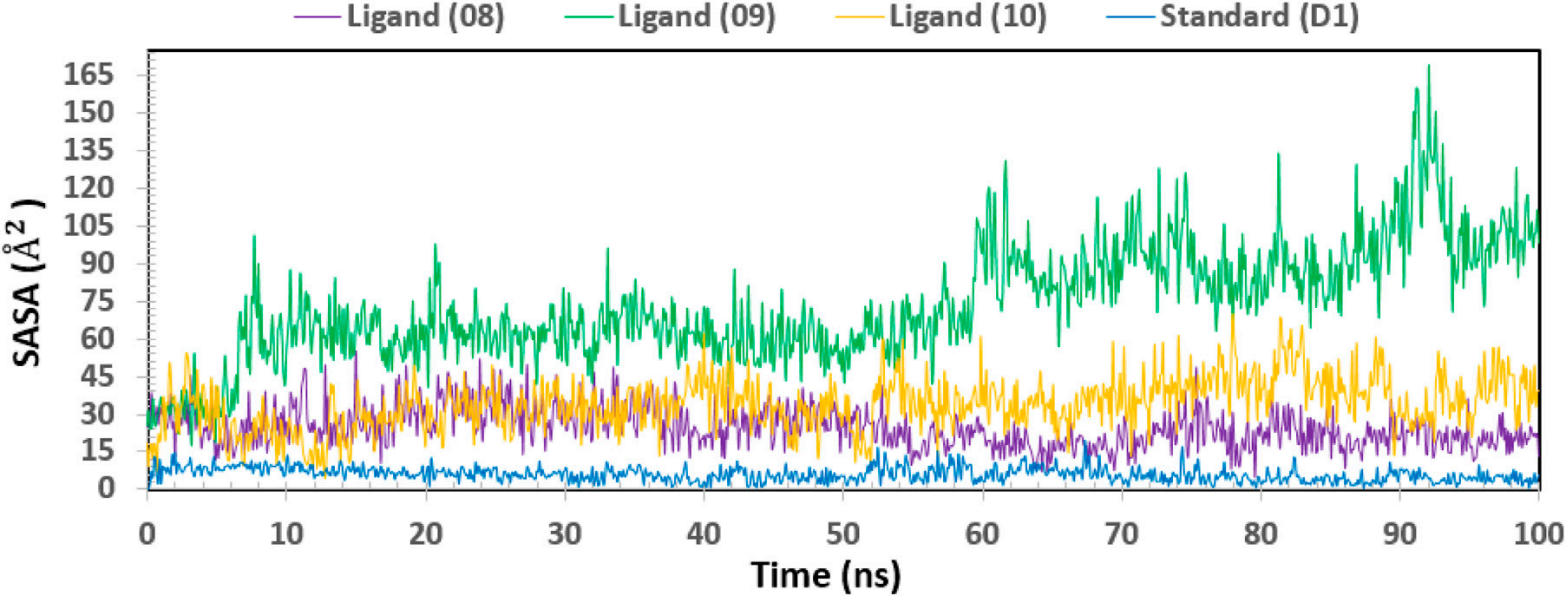
Showing the graph of solvent accessible surface area (SASA) of the protein–ligand complex for 100 ns simulation. The SASA value of the selected four compounds ligand (08), ligand (09) ligand (10) and standard (D1) in complex with the breast cancer represented by a purple, green, orange, and blue color, respectively.

Biological macromolecules’ Solvent-Accessible Surface Area (SASA) affects both their structure and functionality. The residues of amino acids on the surface of proteins are usually hydrophobic or hydrophilic molecules that interact with other molecules and ligands to generate active sites and/or provide information about the behavior of molecules and protein-ligand complexes in various solvents. Thus, Figure 12 displays the SASA value of the protein upon interaction with Ligand Nos. 08, 09, 10, and D1. The complex system’s SASA value, which was found to be a mean between 05 and 165 Å^2^, demonstrated a high level of interaction between an amino acid residue and the selected molecule (Bogatyreva and Ivankov, 2008).

### Ligand-protein contacts evaluations

**FIGURE 13:**
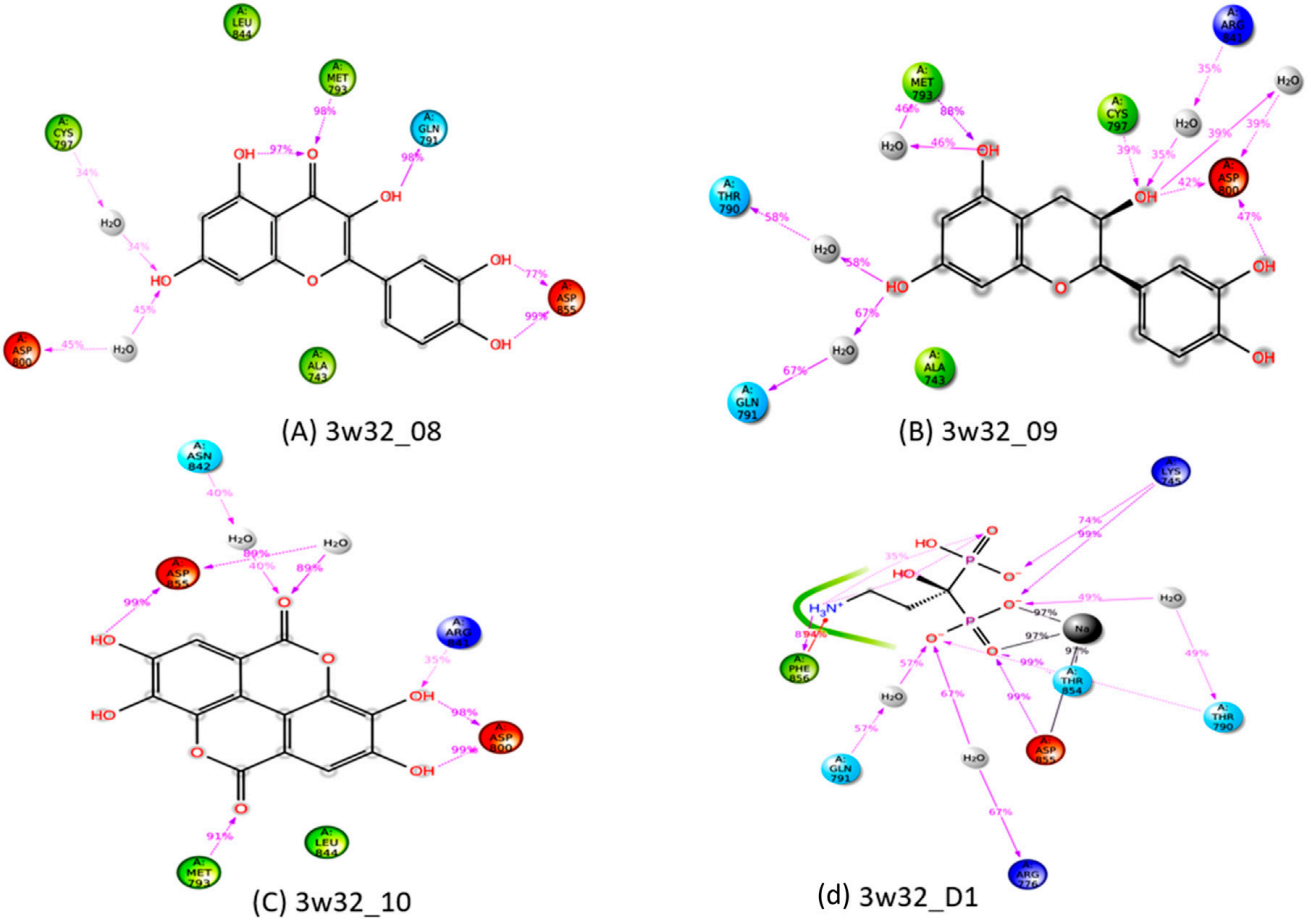
Ligand-Protein interactions diagram after simulations of 100ns: **(A)** complex of ligand 08 and protein 3w32, **(B)** complex of ligand 09and protein 3w32, **(C)** complex of ligand 10 and protein3w32, and **(D)** complex of standard D1 and protein 3w32.

One of the most crucial and significant discoveries made during the SID monitoring for the 100 ns MD simulation is the evaluation of the ligand and protein contract. Following simulations, the four selected ligands (Ligand 08, Ligand 09, Ligand 10, and Standard D1) and the 3w32 proteins identified in breast cancer are shown in their interaction diagrams in Figure 13. At the active sides of ASP855, MET793, GLN791, ASP800, and CYS797, the ligand 08 interacts with protein 3w32 to form numerous (more than two) interactions with simulation times of 99%, 99%, 98%, 45%, and 34% of specific interactions sustained by the multiple contacts illustrated in Figure 13A. When ligand 09 and protein 3w32 are coupled, as Figure 13B illustrates, they interact with the active sides of MET793, GLN791, THR790, ASP800, CYS797, and ARG841 with 88%, 67%, 58%, 42%, 39%, and 35%, respectively. The active sides of ASP800, ASP855, MET793, ASN842, and ARG841 interact at 99%, 99%, 92%, 40%, and 35% when ligand 10 contracts with protein 3w32 in Figure 13C. The active sides LYS745, THR854, ASP855, PHE856, GLN791, ARG776, and THR790 interact at 99%, 99%, 99%, 94%, 57%, 67%, and 49% when the standard D1 contracts with protein 3w32, as shown in Figure 13D.

### Evaluation of ADME properties

The development of new medications relies heavily on DMPK analysis. The acronym ADME stands for “absorption, distribution, metabolism, and elimination,” which describes the steps taken by the body to digest and eliminate a medicine (Pellegatti, 2012). Research like this is useful for gauging the possible effectiveness of a drug (Li, 2001). The first is distribution, which describes the speed and extent of medication delivery to various parts of the body following administration. The second component is absorption, which defines the rate and amount of medication absorption into the bloodstream following administration (Yang et al., 2017). The term “elimination” describes how quickly and efficiently a medicine is absorbed into the bloodstream. Metabolic rate (Zhang and Tang, 2018), action mechanism (Currie, 2018), metabolite structure (Guengerich, 2011), and therapeutic efficacy or safety (Smith, 2011) are all aspects of a medicine’s metabolism. The negative and side effects of drugs are commonly referred to as “toxicology”. Table 6 displays the methodology used to acquire data on the medicine’s ADME profile from a computational forecasting internet resource. All possible therapeutic choices are quickly absorbed by humans due to their intestinal absorption rate of about 85%. For therapeutic chemicals to cross the blood-brain barrier, all of them have been found to be subcellularly localized in mitochondria. With values ranging from −1.381 to −6.773, all of the ligands were quite soluble in water; however, ligand 08 had the lowest aqua solubility value and ligand 09 had the highest. Neither a substrate nor an inhibitor for CYP 2D6 could be located.

**TABLE 6:** Data of ADME properties.

	Absorption			Distribution		Metabolism		Excretion	
S/N	Water solubility (Log mol/L)	Human Intestinal Absorption (%)	Caco-2 Permeability +/−	VDss (human) (log L/kg)	BBB Permeability (log BB)	CYP 2D6 Inhibitor	CYP 2D6 Substrate	Total Clearance (mL/min/kg)	Renal OCT2 substrate
01	−5.668	96.351	1.223	−0.048	0.705	No	No	0.151	No
02	−7.498	93.119	1.203	0.660	0.683	No	No	0.403	No
03	−3.181	86.684	0.335	0.375	−1.272	No	No	0.537	No
04	−3.040	74.29	0.032	1.274	−0.939	No	No	0.477	No
05	−1.381	13.831	−0.395	−0.998	−0.788	No	No	0.810	No
06	−0.660	71.748	0.603	−1.013	−0.163	No	No	0.722	No
07	−6.092	85.891	1.101	−0.016	1.222	No	No	2.188	No
08	−1.423	0.000	−0.240	−0.418	−1.017	No	No	0.895	No
09	−6.773	94.464	1.201	0.193	0.781	No	No	0.628	No
10	−6.267	95.124	1.231	0.192	0.689	No	No	−0.050	No
11	−2.504	95.277	1.184	0.034	−0.299	No	No	0.730	No
12	−3.444	95.824	1.251	−0.956	0.095	No	No	0.584	No
13	−1.377	21.510	−0.249	0.148	−0.943	No	No	0.626	No
14	−2.925	47.999	0.242	1.846	−1.688	No	No	0.394	No
15	−2.891	0.000	−1.668	0.310	−2.707	No	No	−0.418	No
D1	−3.258	100.000	0.521	1.163	−1.531	No	No	−0.411	No

### Toxicity analysis

The possible negative effects of a substance that resembles a drug on living things are referred to as “toxicity” (Smith, 2011). The toxicity of the chosen ligands in Table 7 has been assessed in both aquatic and non-aquatic environments. Furthermore, none of the chemicals have any negative effects on the environment and are safe for ingestion by humans. The industrial compounds’ acute oral toxicity varied greatly, from 1.156 kg/mol to 2.541 kg/mol. With the exception of ligands 05 and 06, all examined ligands have been confirmed to be carcinogen-free, indicating that the chemical described here does not pose a danger of cancer to living things. But it has also been shown that the ligands mentioned are non-toxic, which means that there is no risk to the environment or human health from them. Due to their lack of skin effects, the necessary ligands can be handled freely in the pharmaceutical business.

**TABLE 7:** Aquatic and non-aquatic toxicity of selected ligands.

Ligand No	AMES toxicity	Hepatotoxicity	Oral rat Chronic Toxicity (mg/kg.bw/day)	Oral rat acute toxicity (LD50) (mol/kg)	Max. Tolerated dose (mg/kg/day)	T. Pyriformis toxicity (log ug/L)	Skin sensitisation
01	No	No	3.060	2.218	0.700	0.285	No
02	No	No	2.963	1.156	0.994	0.263	No
03	No	No	2.021	2.423	0.814	0.273	No
04	No	No	2.494	1.513	0.567	0.285	No
05	Yes	No	2.461	2.164	0.496	0.367	No
06	Yes	No	2.432	1.898	−0.296	0.195	No
07	No	No	3.897	1.214	1.896	0.285	No
08	No	No	2.612	2.471	0.499	0.288	No
09	No	No	2.500	2.428	0.438	0.347	No
10	No	No	2.698	2.399	0.476	0.295	No
11	No	No	4.417	2.541	0.569	0.285	No
12	No	No	4.277	2.396	0.58	0.285	No
13	No	No	3.977	2.373	0.613	0.285	No
14	No	No	4.417	2.541	0.569	0.285	No
15	No	No	5.113	2.481	0.4	0.285	No
D1	No	No	4.417	2.541	0.569	0.285	No

## Discussion

Specifically, breast cancer refers to a malignant tumour that develops from cells within the breast. Among female-identifying cancers, it ranks first globally. Several factors, including heredity, lifestyle choices, and environmental exposure, increase the likelihood of breast cancer developing. Nevertheless, investigations into the underlying cellular and molecular processes of breast cancer’s initiation, development, and metastasis are continuing. However, there are currently no viable alternatives to antiviral drugs that can combat the virus responsible for breast cancer. The proteins linked to breast cancer (PDB ID 3w32) play a crucial role in the advancement of the disease, according to recent discoveries. The goal of this study is to find a new and effective antiviral medication that can target the 3W32 proteins that are found in breast cancer. The experimental protein structure of 3w32 in the presence of many inhibitory drugs was initially sought after by searching the protein database. The fifteen compounds were selected from the leaf extract of Mangifera indica during the *in silico* investigation.

The assessment of the anti-viral and anti-cancer capacities, among other PASS predictive features, is shown in Supplementary Table S4. Activities against viruses and tumours were found in ligands 08, 09, and 10 (Yap et al., 2021b). We evaluated the pharmacokinetics of the four compounds using Lipinski’s rules five (RO5) for molecules, and we found that they all had the desired ADME properties. Table 4 shows that all three of the selected ligands maintained RO5 levels and had excellent pharmacokinetic properties. Additional evaluation of the chemical’s harmful effects on humans and animals has been conducted using the toxicity features of the molecule with good ADME properties. None or very little toxicity was seen with the three ligands selected for the study (Aljahdali et al., 2021a).

A computational DFT-based quantum mechanical simulation was used to investigate and optimize the ligand form. The DFT-optimized geometry was recovered. The FMO-based HOMO-LUMO energy gap was computed for a more thorough evaluation of the ligands’ chemical activity. All of the ligands had HOMO-LUMO gap energies that were higher than 3.50 eV. Their low reactivity is consistent with their bioactivity, as seen in (Bouback et al., 2021). Molecular docking simulations were used to perform additional testing on the sixteen compounds that were selected, including standard (D1) and ligands 01–15. The docking scores produced by ligand 08, ligand 09, ligand 10, and standard (D1) against protein 3w32 were −8.5 kcal/mol, −8.4 kcal/mol, −8.5 kcal/mol, and −6.3 kcal/mol, respectively, as shown in Table 5. Not only does this docking score surpass the norm (>-6.0 kcal/mol) (Kodical et al., 2020), but we also found that the scores of the three selected ligands were greater than the standard (D1).

A molecular dynamics simulation is used to confirm the stability of a protein when it is bound to a ligand. Not only that, it can measure the stability and rigidity of protein-ligand complexes in a specific synthetic setting, like the human body (Alam et al., 2021) (Aljahdali et al., 2021b). By comparing the RMSD values of different complex systems, we can see which compounds are the most stable, and by comparing the RMSF values of different protein-ligand complexes, we can see how compact they are (Krupanidhi et al., 2021). Using the Ca atoms of the protein-ligand complexes, the RMSD of the system was calculated, validating the small protein changes. By calculating the protein’s fluctuation using the RMSF value, we can see that the chemicals are stable for the target protein and that the complex system has low variation. With a smaller Rg value indicating tremendous compactness and a bigger value showing the disassociation of the ligands from the protein, all of the ligands display a greater Rg value (Elebeedy et al., 2021). A smaller SASA value indicates a less stable structure, which is indicative of a more compressed complex of water molecules and amino acid residues (Mahmud et al., 2021; Mahmoud et al., 2021). Results showed that the three selected ligands all had optimal Rg and SASA values. Following the evaluation of the three ligands chosen based on different qualities, which yielded a range of outcomes, the chemical has been chosen for additional research utilizing a number of wet lab-based experimental approaches.

To expedite the process of discovering new medication candidates, computational drug design enables scientists to foretell the interactions between chemicals and biological targets. Efficiently screening large chemical libraries, optimizing molecular structures, and lowering experimental costs are all made possible by this. It boosts the chances of success in subsequent experimental phases and speeds up the discovery process by simulating interactions.

## Conclusion

An increasingly important, effective, and external method for finding inhibitory molecules against a particular target protein is computer-aided drug design. This work reports on the rapid and effective identification of novel natural inhibitors of cancer proteins through the application of CADD. Ligands 10, 8, and 12 emerge as the top-performing candidates due to their superior binding affinities and high numbers of interactions, with hydrophobic interactions playing a key role in enhancing their binding efficiency. Ligands 9 and 13 also show promise, although they are slightly less effective than the top performers. In contrast, ligands with fewer total bonds and weaker binding affinities, such as ligands 2, 7, and 11, are less favourable for further consideration. In addition, these compounds (Ligands 10, 8, and 12) have the potential to inhibit the activity of breast cancer cells and prevent the replication of ASP855, MET793, GLN791, ASP800, and CYS797 residues by ligand 08, MET793, GLN791, THR790, ASP800, CYS797, and ARG841 residues by ligand 09, and ASP800, ASP855, MET793, ASN842, and ARG841 residues by ligand 10 of 3w32 protease into the human host cell. Next, Ligands 1, 2, 9, 10, 12, and D1 are top candidates due to excellent absorption, balanced distribution, and moderate clearance. Ligands 8 and 15 are poor performers with zero absorption and low distribution. Ligand 7 shows potential for CNS-targeted therapies with high BBB permeability but may require adjustments due to rapid clearance. Further optimization is needed for ligands with extreme values like 5, 6, 8, and 15. However, Ligands 10, 8, and 12 emerge as the top-performing candidates for breast cancer due to investigation of their superior binding affinities, quantum descriptors and strong interactions, making them promising options for further development and investigation.

## Data Availability

The original contributions presented in the study are included in the article/Supplementary Material, further inquiries can be directed to the corresponding author.

## Abbreviations

ADME, Absorption, Distribution, Metabolism, and Excretion; ADMET, Absorption, Distribution, Metabolism, Excretion, and Toxicity; CADD, Computer-Aided Drug Design; DFT, Density Functional Theory; HOMO, Highest Occupied Molecular Orbital; LUMO, Lowest Unoccupied Molecular Orbital; C.B, Breast Cancer; CRT, Chemo-Radiation; RMSD, Root Mean Square Deviation; RMSF, Root Mean Square Fluctuation; MD, Molecular Dynamics; MEP, Molecular Electrostatic Potential; NMR, Nuclear Magnetic Resonance; PASS, Prediction of Activity Spectra for Substances; PK, Pharmacokinetics; QM, Quantum Mechanics; RT, Radiation Therapy; SID, Simulation Interaction Diagram; SASA, Solvent Accessible Surface Area; DMPK, Drug Metabolism and Pharmacokinetics; BBB, Blood-Brain Barrier; CYP 2D6, Cytochrome P450 2D6 (a liver enzyme involved in drug metabolism); OCT2, Organic Cation Transporter 2; LD50, Lethal Dose, 50% (the dose required to kill half the members of a tested population); bw, Body Weight; 3w32, PDB ID of the protein structure used in the study; PDB, Protein Data Bank; AMES, Ames Test (a test used to assess mutagenicity); T. Pyriformis, *Tetrahymena* Pyriformis (a ciliate protozoan used in toxicity testing).
