# Supplementary material for: Chemoinformatics analysis of Mangifera indica leaves extracted phytochemicals as potential EGFR kinase modulators
Source: Front Chem. 2025 Mar 24;13:1524384. doi: 10.3389/fchem.2025.1524384 (PMC11973374; doi:10.3389/fchem.2025.1524384)
Supplement: Supplementary file 1 [file DataSheet1.ZIP › Data/MD Simulation/Simulation Results Analysis/Presentation1.pptx]

## Slide 1
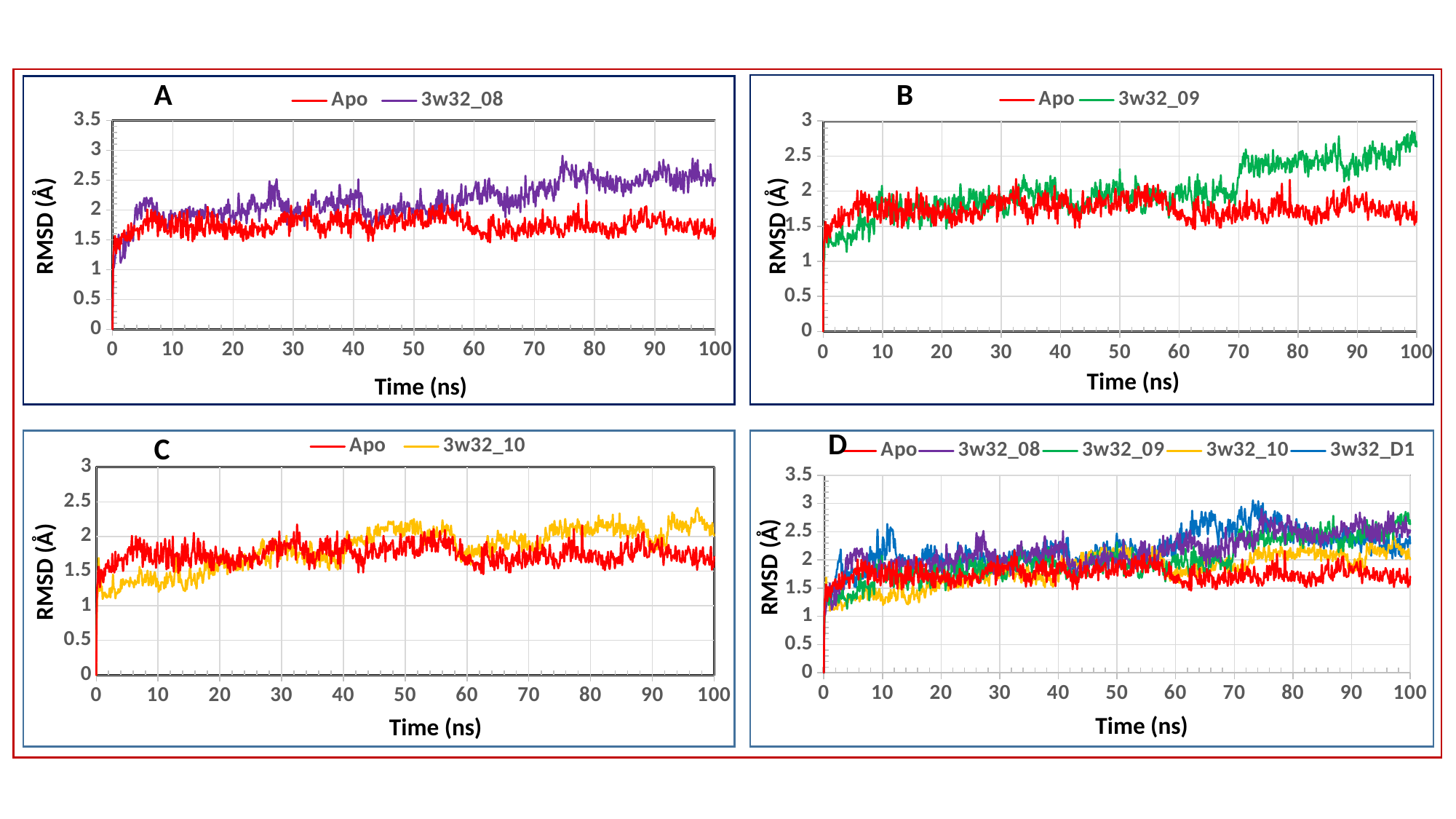

### Chart
| Category | Apo | 3w32_08 |
|---|---|---|A
B
### Chart
| Category | Apo | 3w32_09 |
|---|---|---|RMSD (Å)
RMSD (Å)
Time (ns)
Time (ns)
### Chart
| Category | Apo | 3w32_08 | 3w32_09 | 3w32_10 | 3w32_D1 |
|---|---|---|---|---|---|D
C
### Chart
| Category | Apo | 3w32_10 |
|---|---|---|
RMSD (Å)
RMSD (Å)
Time (ns)
Time (ns)

## Slide 2
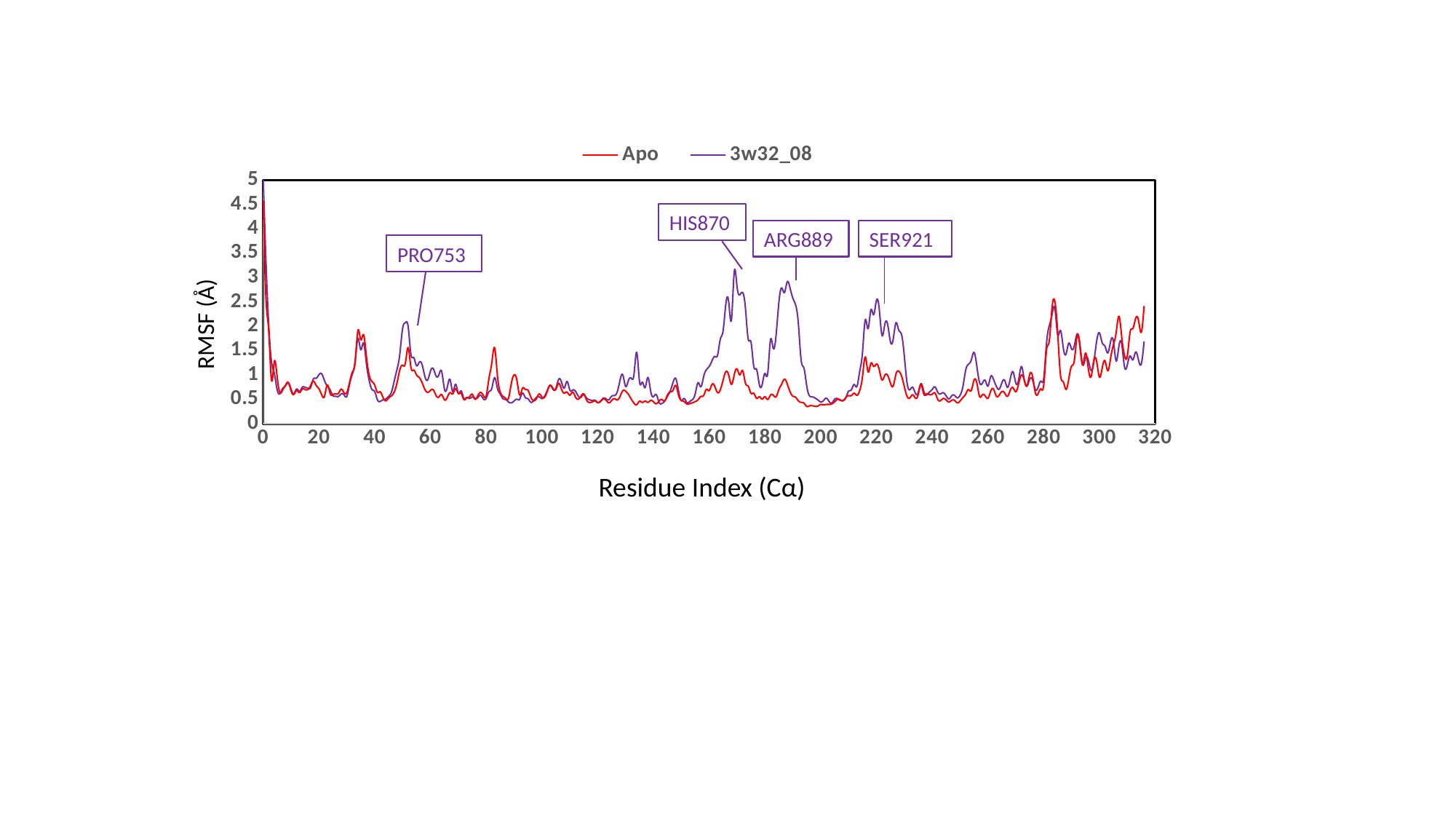

### Chart
| Category | Apo | 3w32_08 |
|---|---|---|HIS870
ARG889
SER921
PRO753
RMSF (Å)
Residue Index (Cα)

## Slide 3
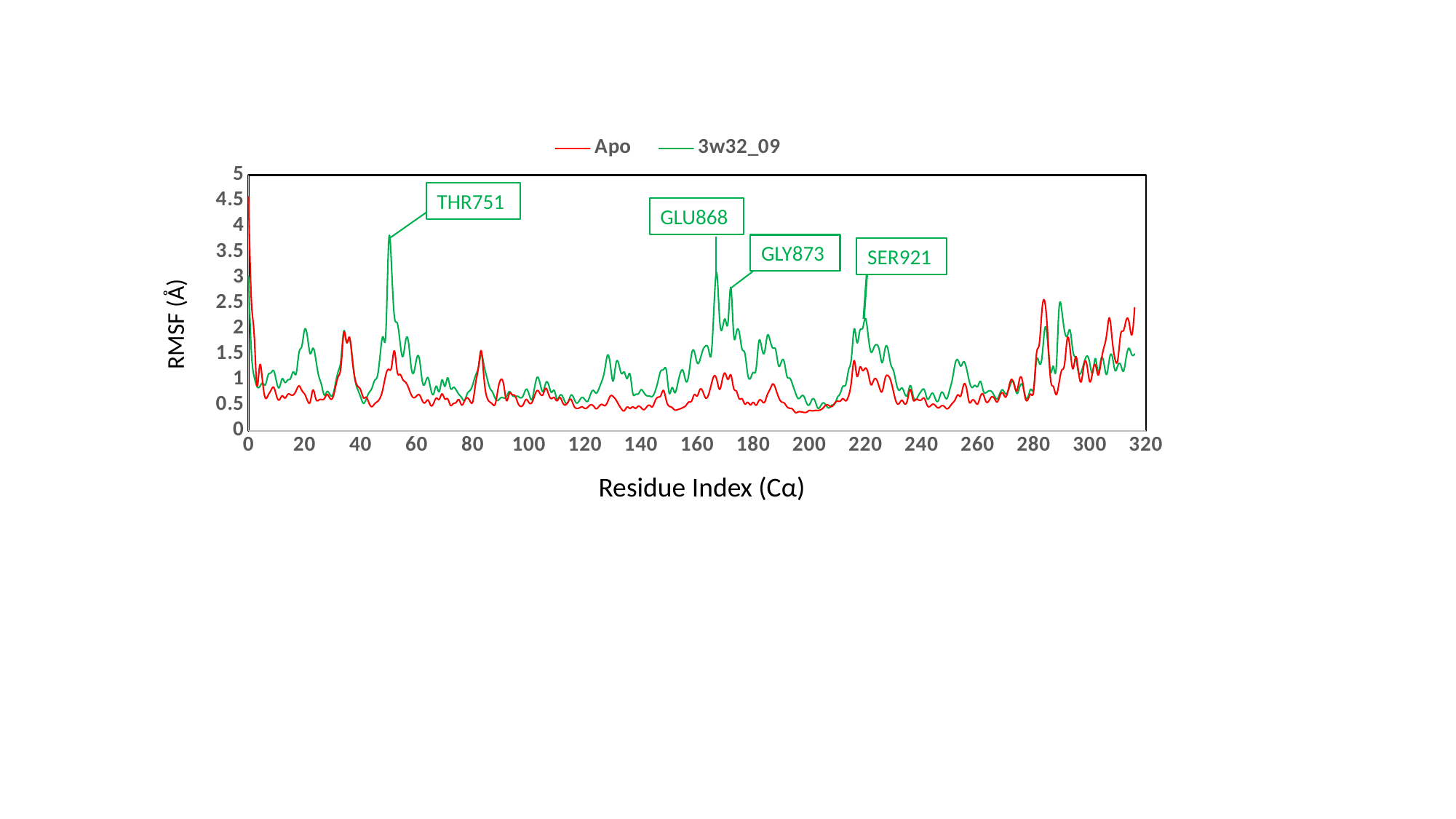

### Chart
| Category | Apo | 3w32_09 |
|---|---|---|THR751
GLU868
GLY873
SER921
RMSF (Å)
Residue Index (Cα)

## Slide 4
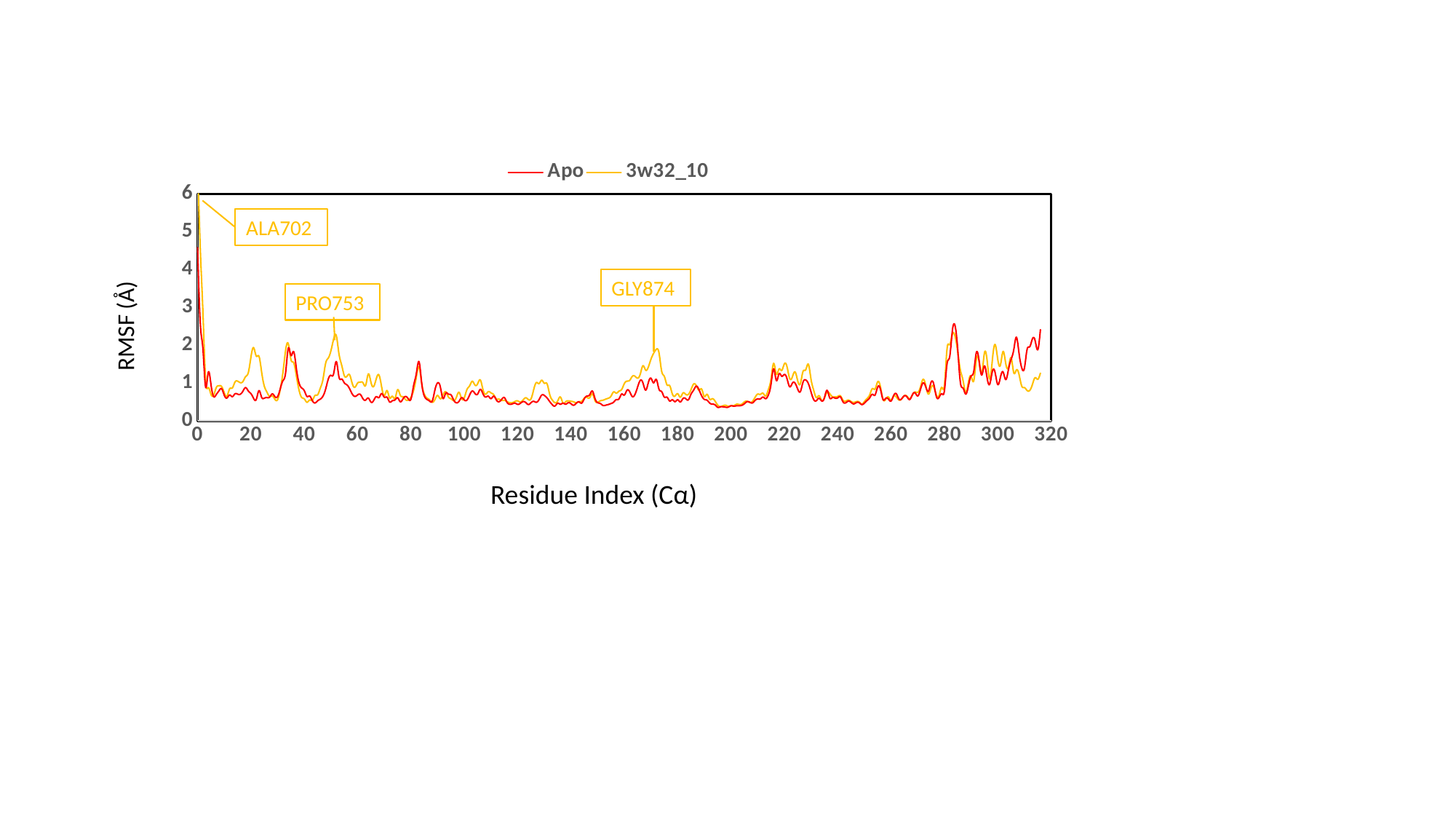

### Chart
| Category | Apo | 3w32_10 |
|---|---|---|ALA702
GLY874
PRO753
RMSF (Å)
Residue Index (Cα)

## Slide 5
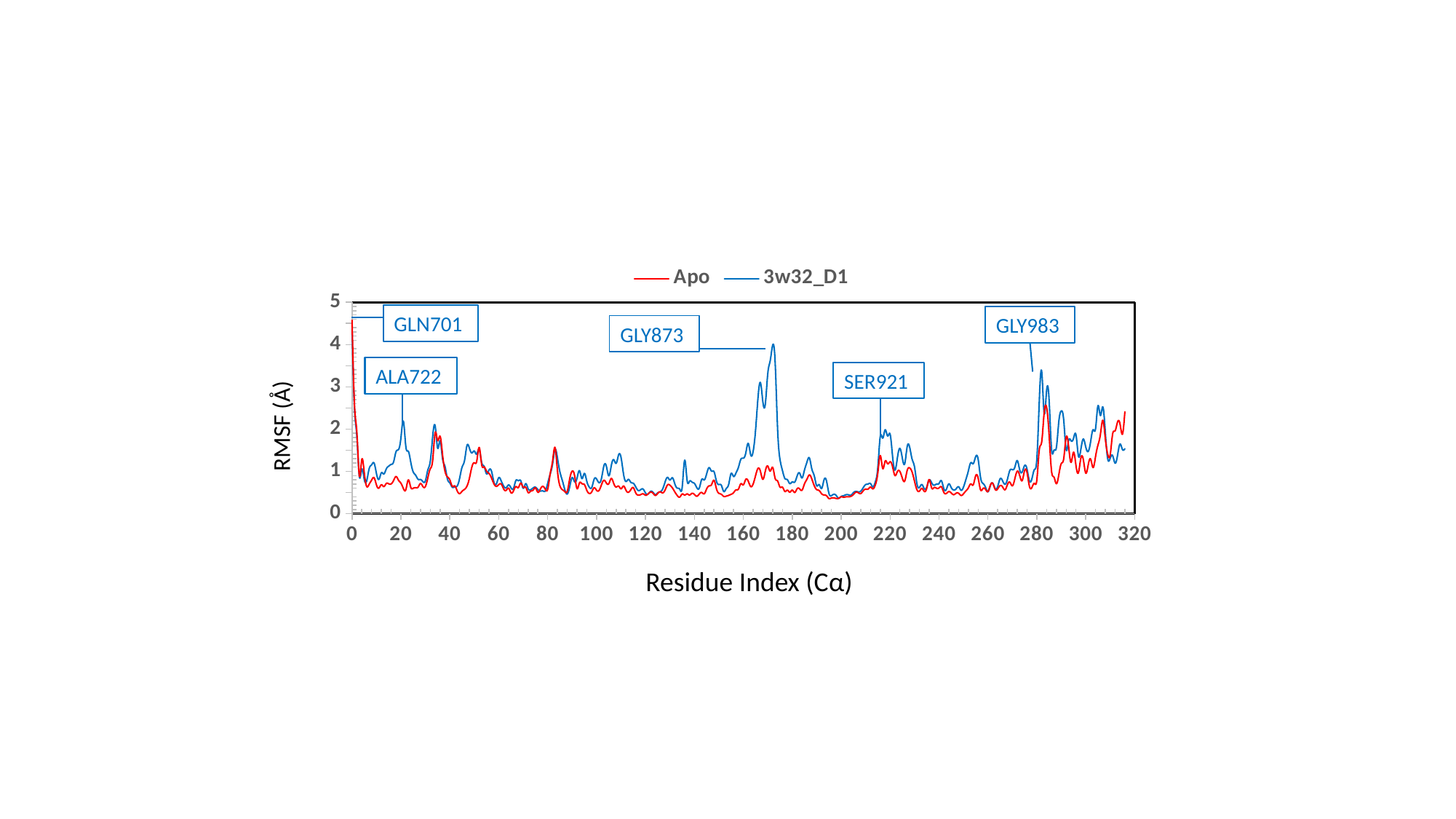

### Chart
| Category | Apo | 3w32_D1 |
|---|---|---|GLN701
GLY983
GLY873
ALA722
SER921
RMSF (Å)
Residue Index (Cα)

## Slide 6
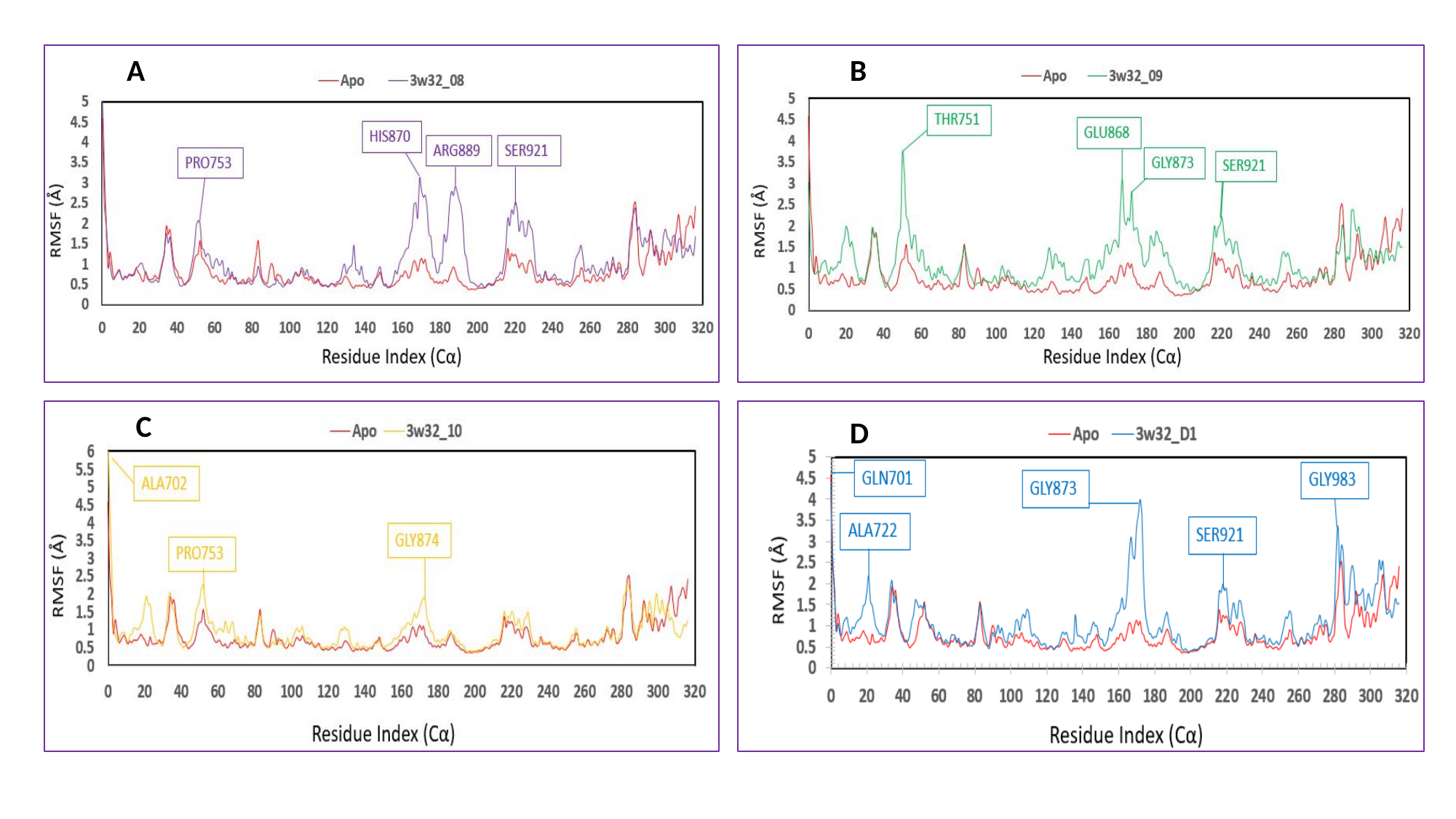

A
B
C
D

## Slide 7
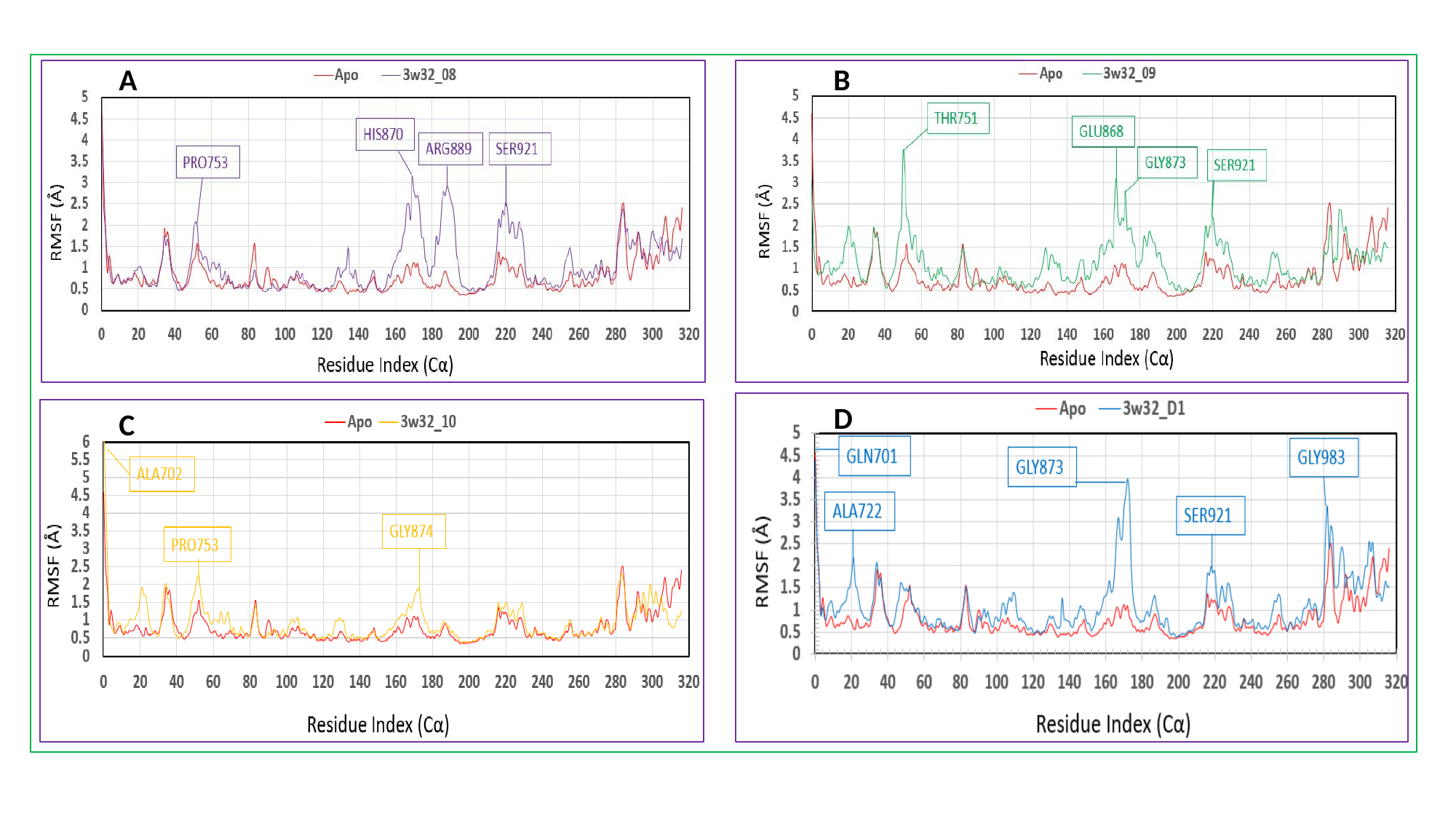

B
A
D
C

## Slide 8
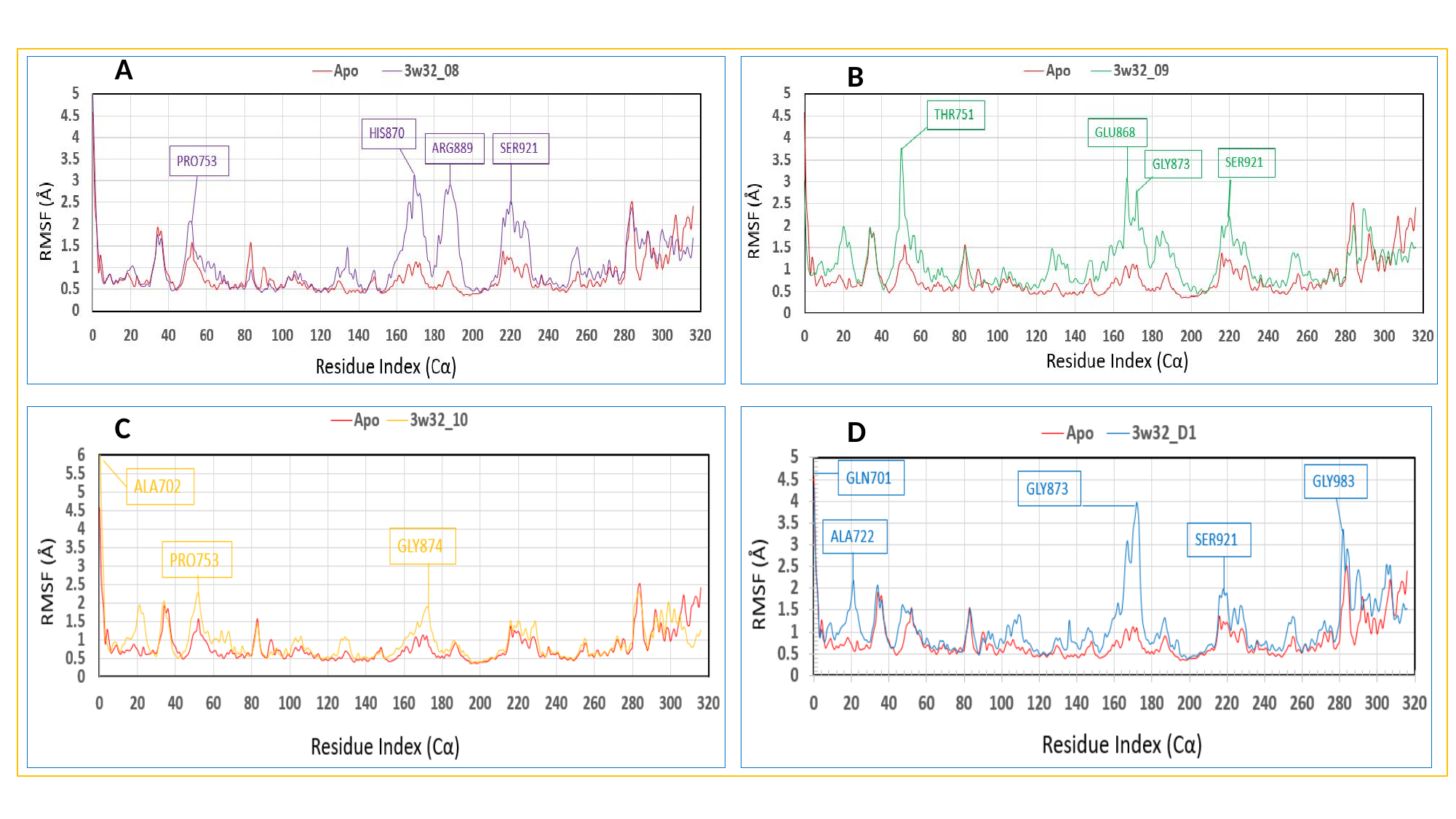

A
B
C
D

## Slide 9
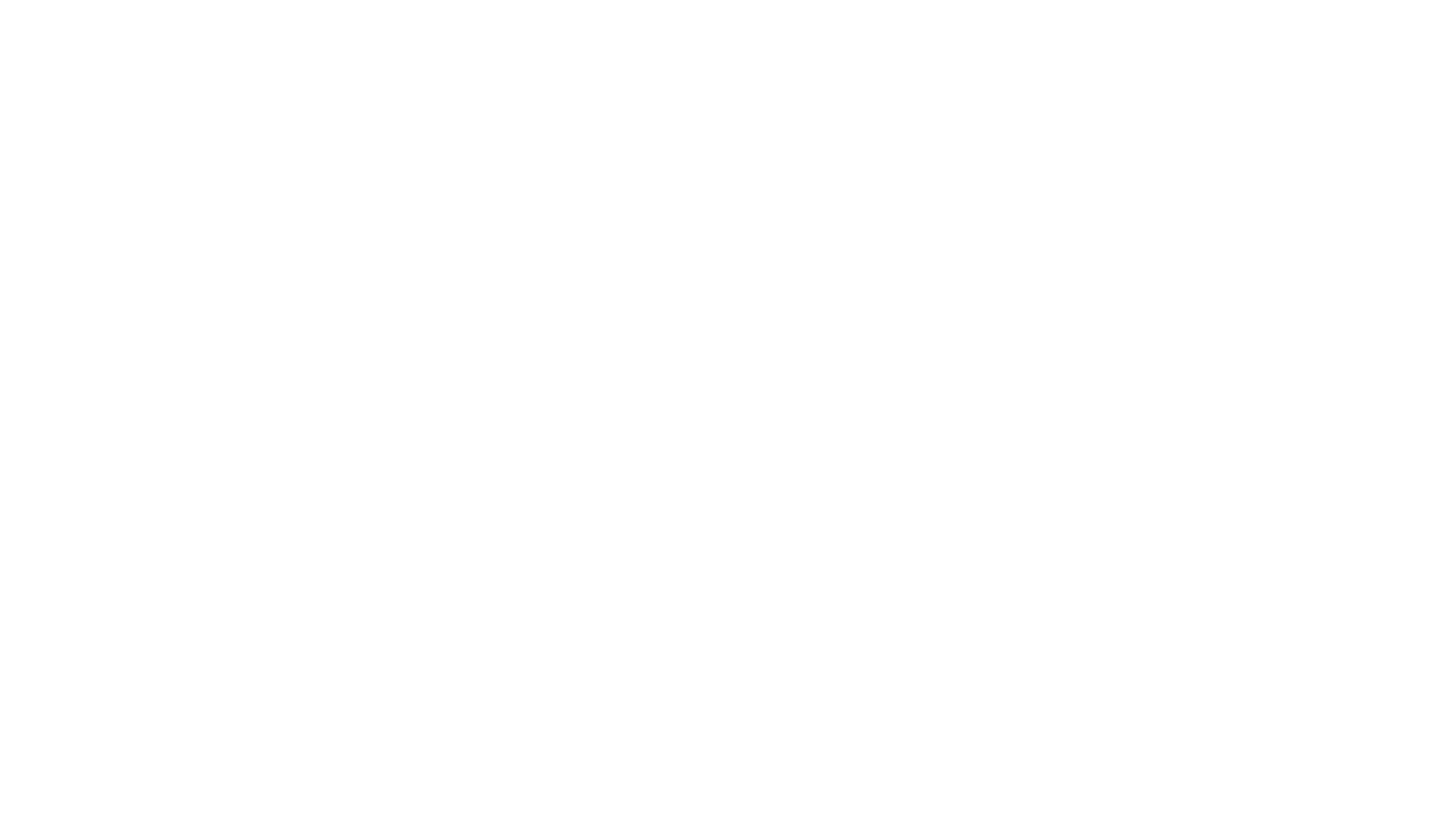

## Slide 10
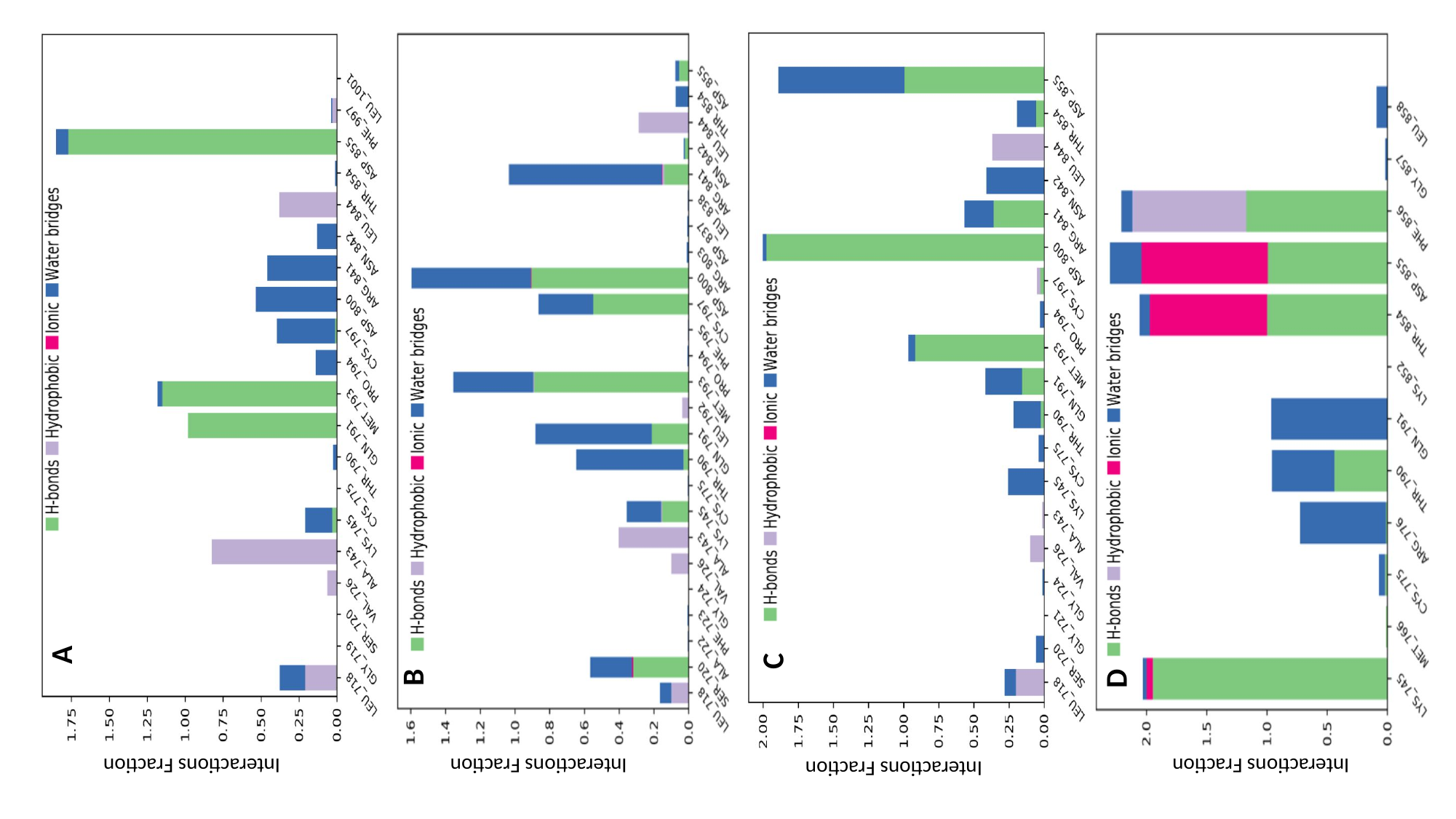

A
C
B
D
Interactions Fraction
Interactions Fraction
Interactions Fraction
Interactions Fraction

## Slide 11
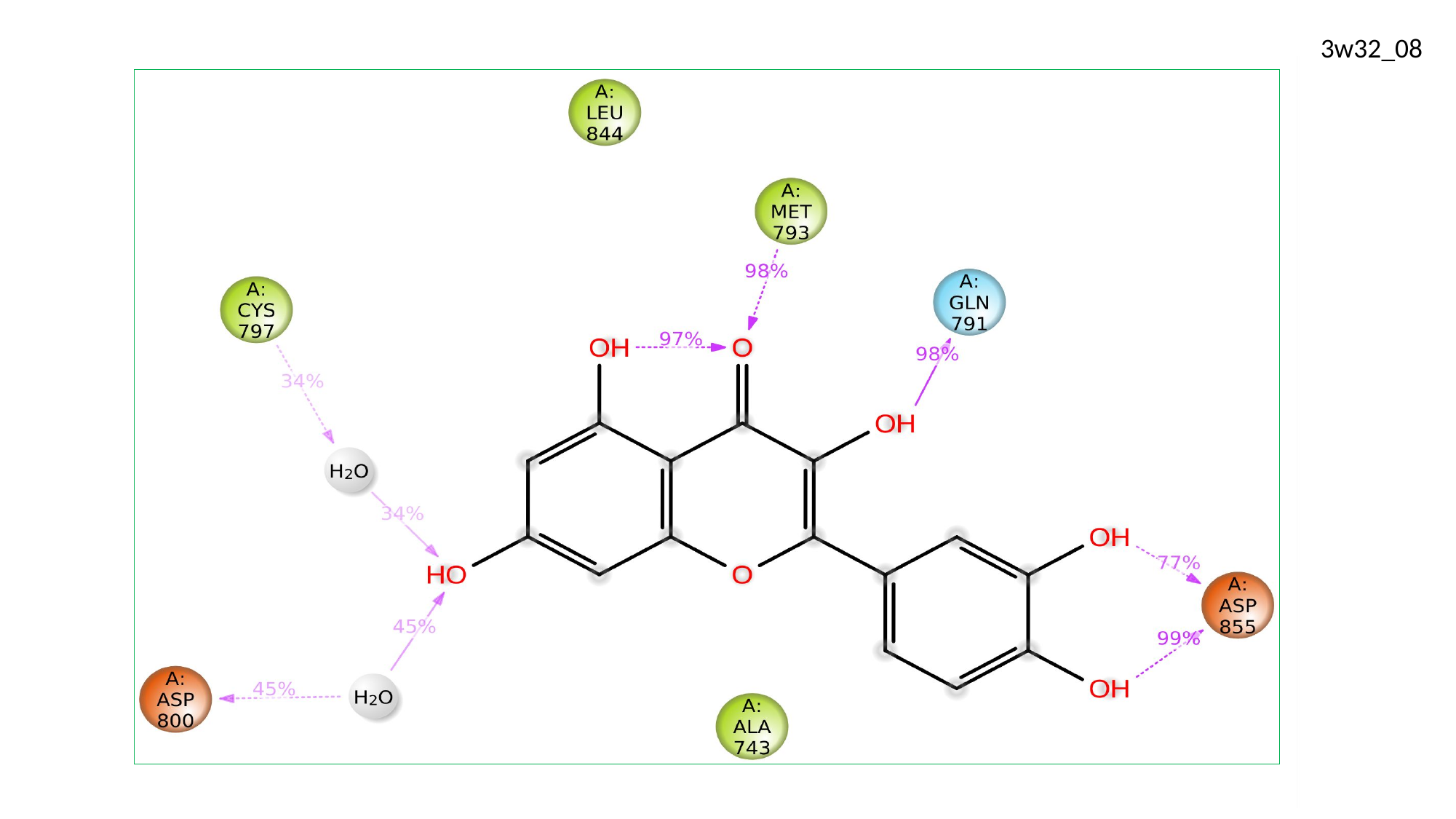

3w32_08

## Slide 12
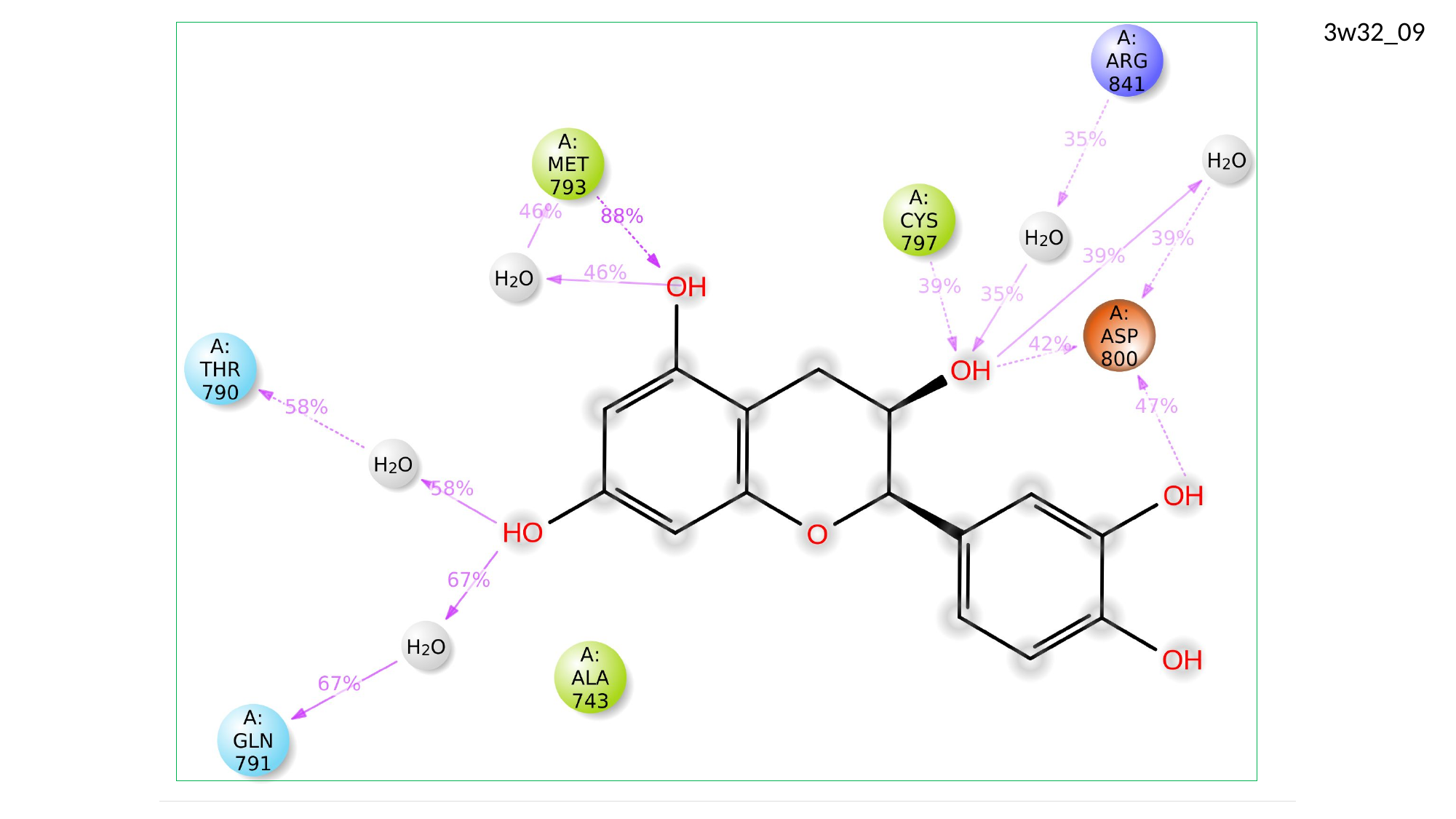

3w32_09

## Slide 13
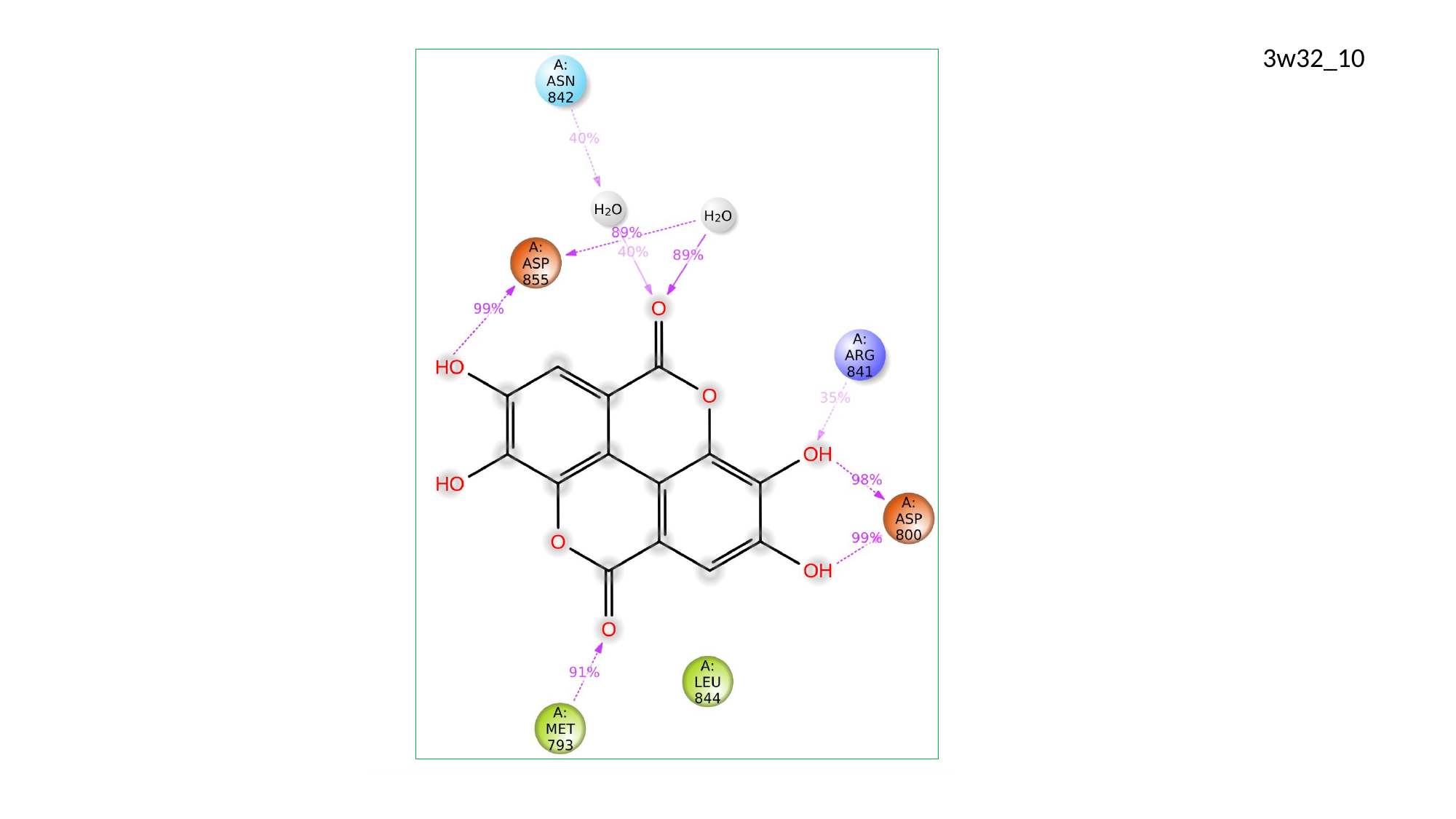

3w32_10

## Slide 14
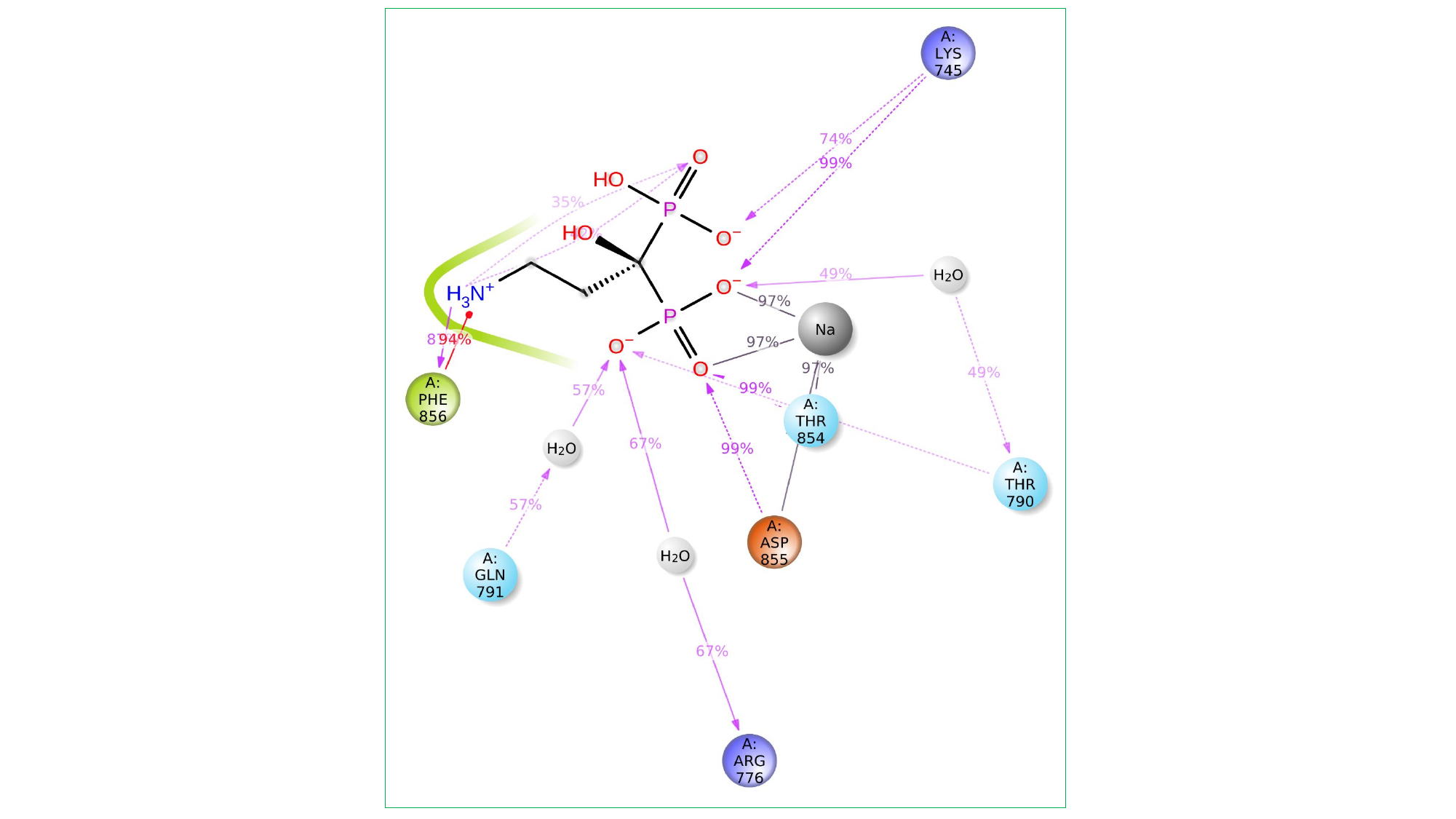

## Slide 15
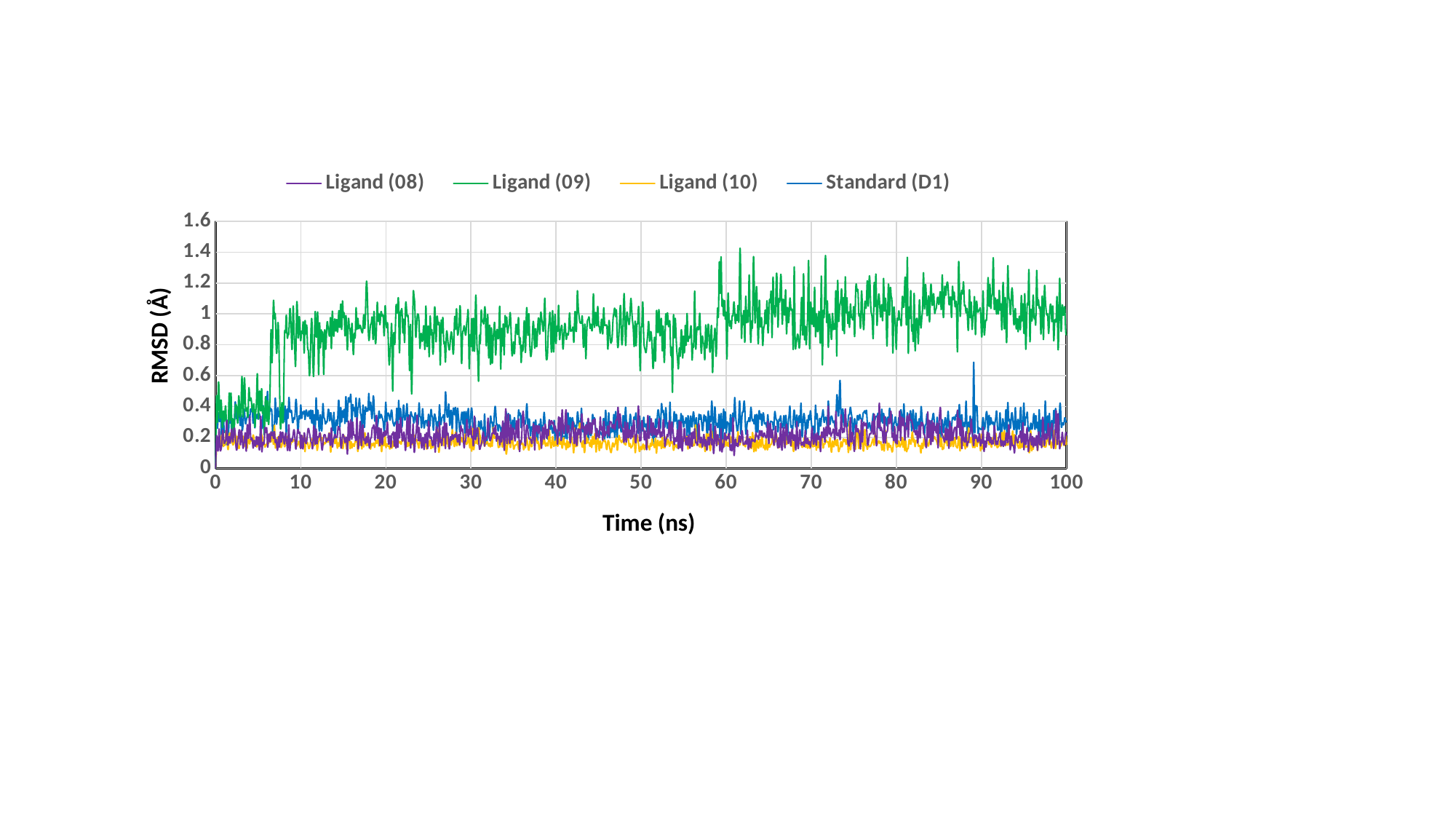

### Chart
| Category | Ligand (08) | Ligand (09) | Ligand (10) | Standard (D1) |
|---|---|---|---|---|RMSD (Å)
Time (ns)

## Slide 16
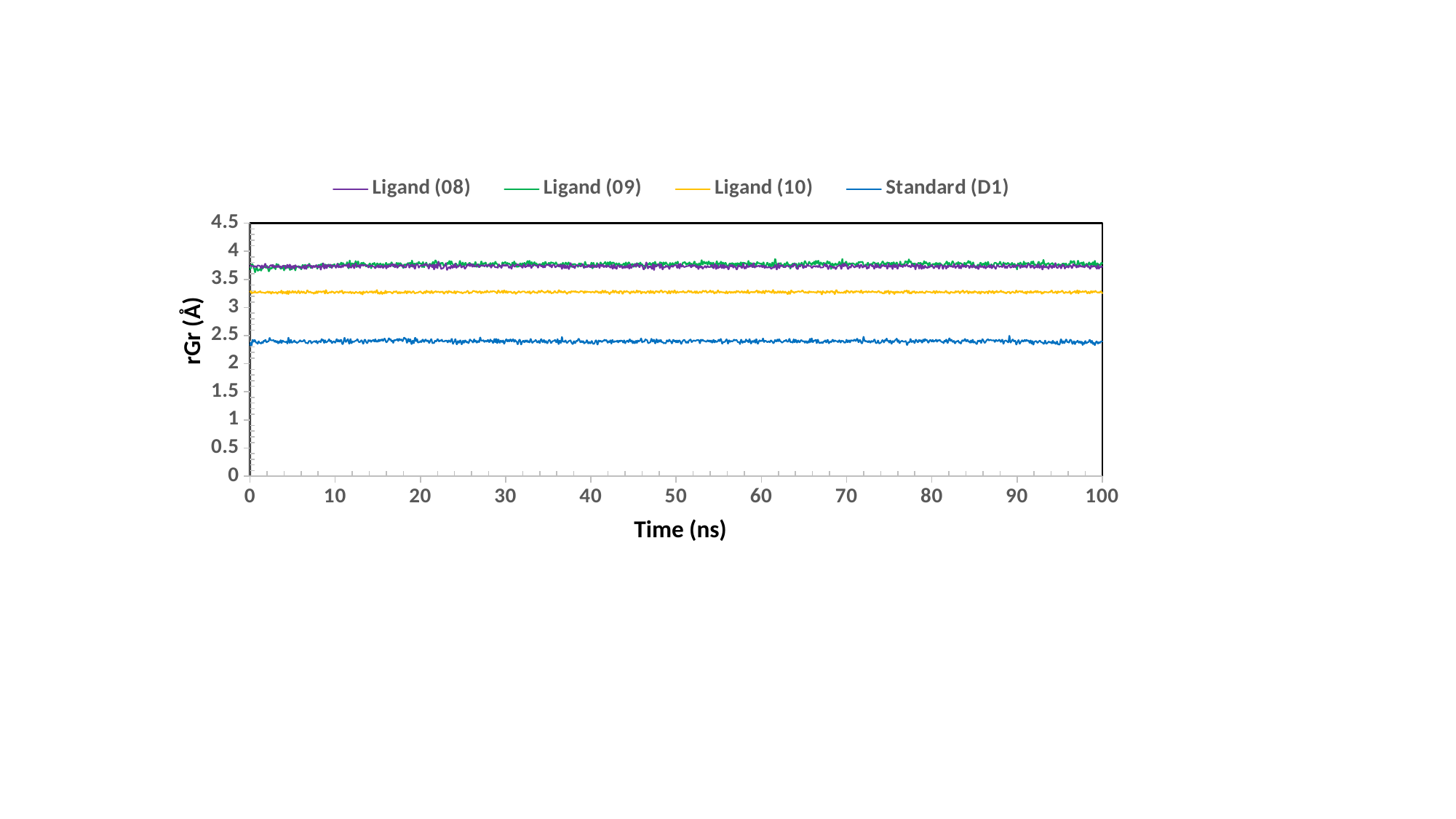

### Chart
| Category | Ligand (08) | Ligand (09) | Ligand (10) | Standard (D1) |
|---|---|---|---|---|rGr (Å)
Time (ns)

## Slide 17
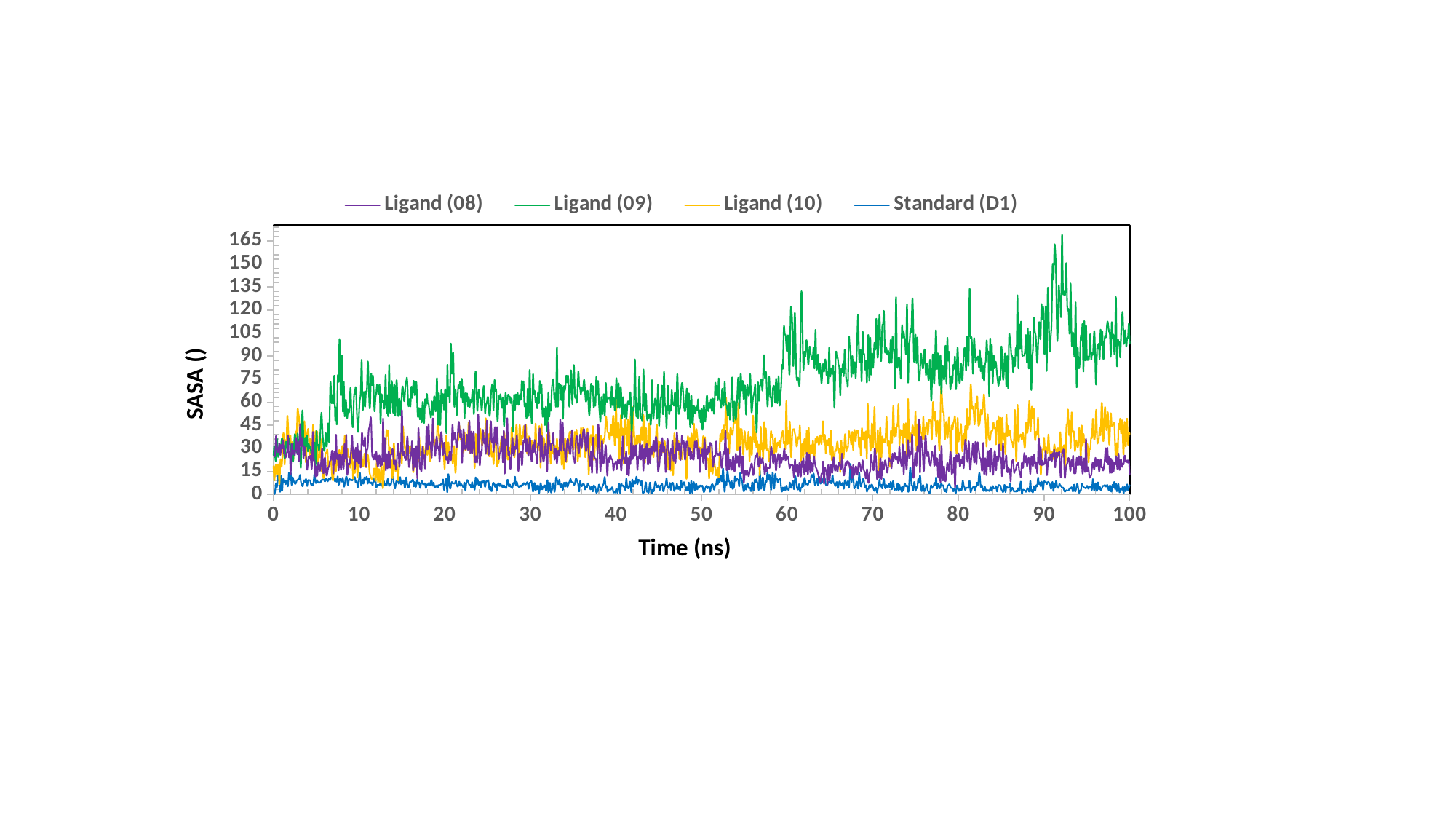

### Chart
| Category | Ligand (08) | Ligand (09) | Ligand (10) | Standard (D1) |
|---|---|---|---|---|Time (ns)

## Slide 18
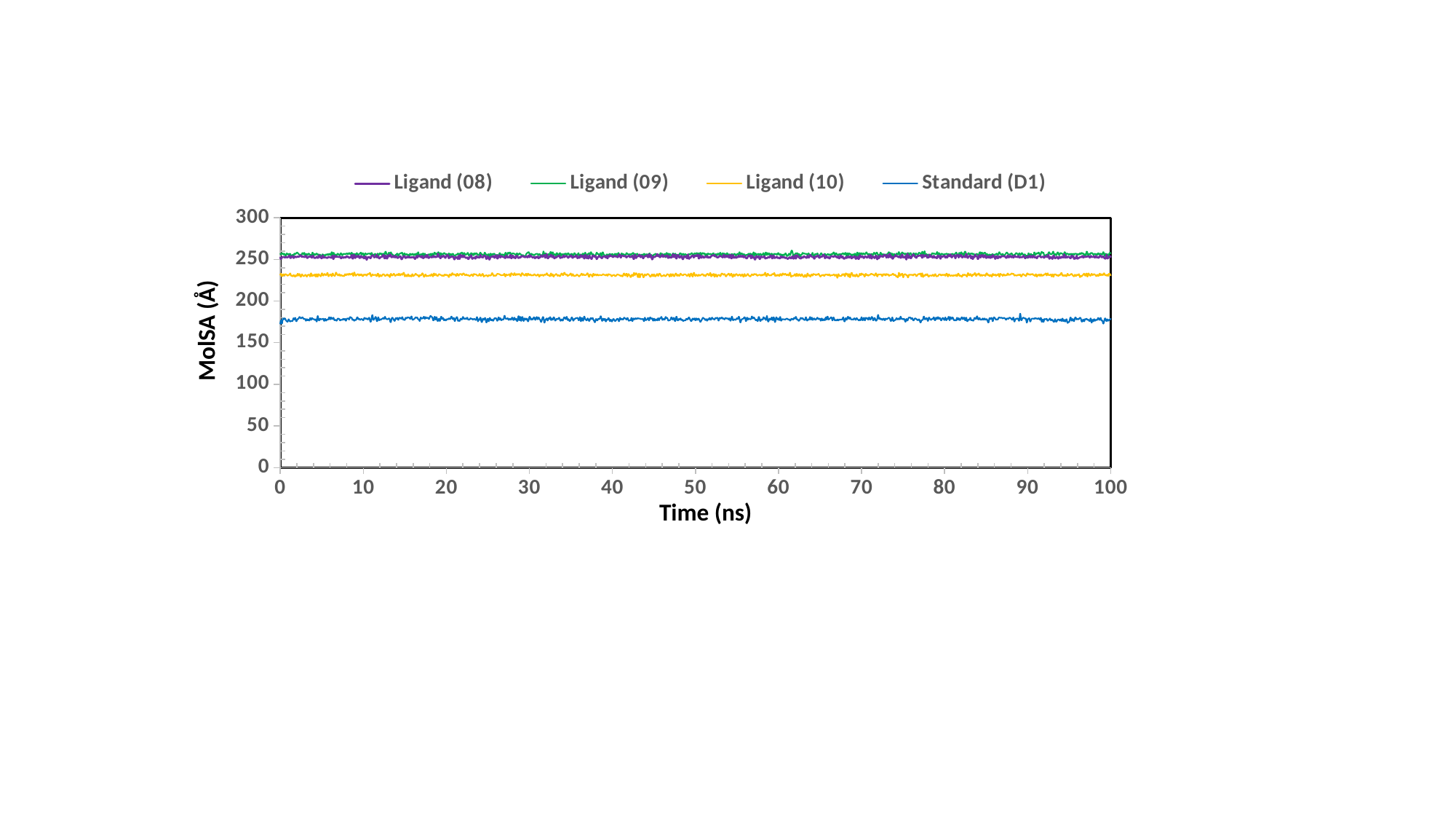

### Chart
| Category | Ligand (08) | Ligand (09) | Ligand (10) | Standard (D1) |
|---|---|---|---|---|MolSA (Å)
Time (ns)

## Slide 19
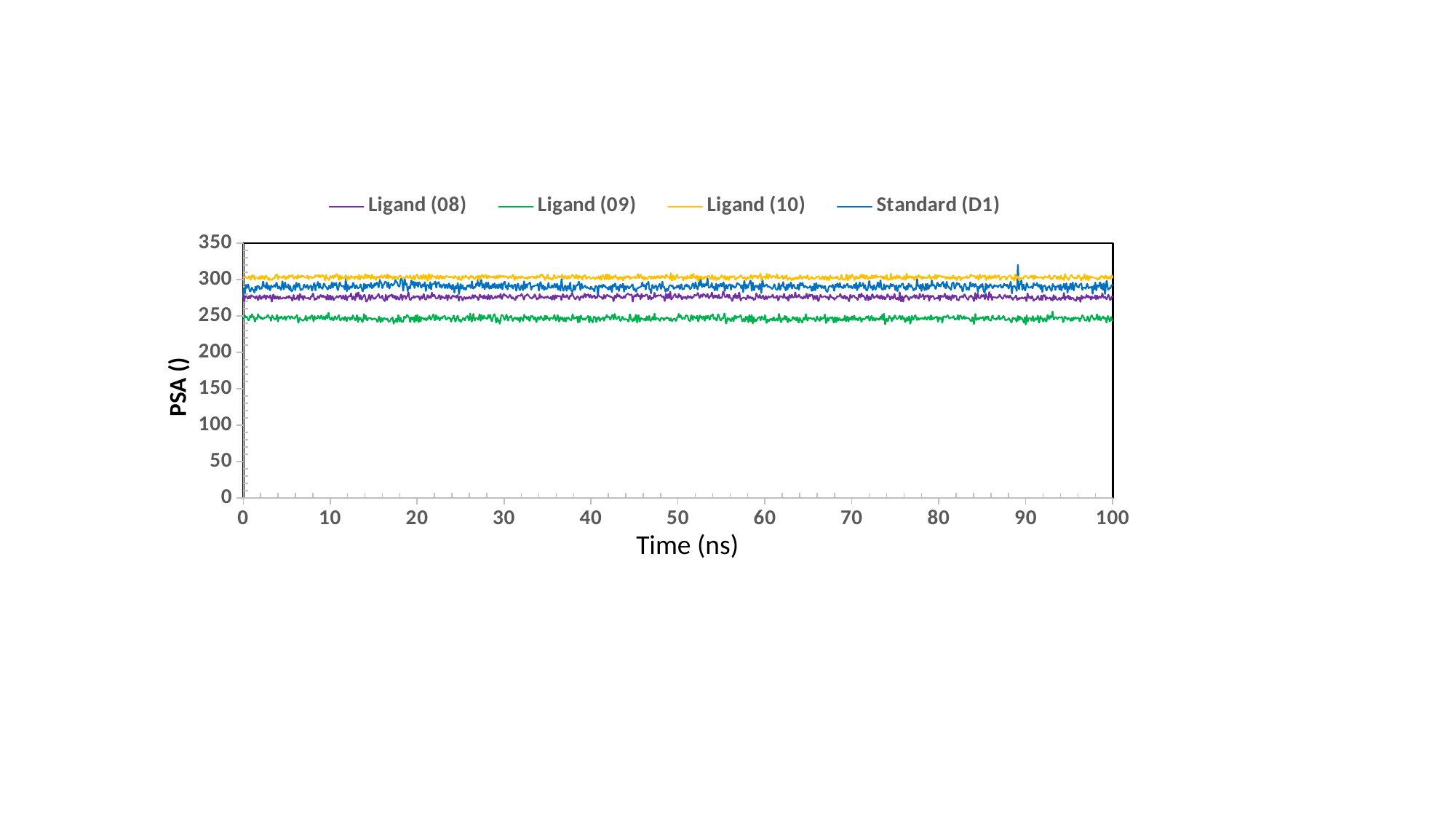

### Chart
| Category | Ligand (08) | Ligand (09) | Ligand (10) | Standard (D1) |
|---|---|---|---|---|Time (ns)
