# Supplementary material for: Chemoinformatics analysis of Mangifera indica leaves extracted phytochemicals as potential EGFR kinase modulators
Source: Front Chem. 2025 Mar 24;13:1524384. doi: 10.3389/fchem.2025.1524384 (PMC11973374; doi:10.3389/fchem.2025.1524384)
Supplement: Supplementary file 1 [file DataSheet1.ZIP › Ligands Information.docx]

**Table: Ligand Information**

| **S/N** | **Name of Ligand** | **PubChem CID** | **Chemical Formula** |
| --- | --- | --- | --- |
| 1 | Gallic acid | 370 | [C_7_H_6_O_5_](https://pubchem.ncbi.nlm.nih.gov/#query=C7H6O5) |
| 2 | Shikimic acid | 8742 | [C_7_H_10_O_5_](https://pubchem.ncbi.nlm.nih.gov/#query=C7H10O5) |
| 3 | Protocatechuic acid | 72 | [C_7_H_6_O_4_](https://pubchem.ncbi.nlm.nih.gov/#query=C7H6O4) |
| 4 | Kainic acid | 10255 | [C_10_H_15_NO_4_](https://pubchem.ncbi.nlm.nih.gov/#query=C10H15NO4) |
| 5 | Norathyriol | 5281656 | [C_13_H_8_O_6_](https://pubchem.ncbi.nlm.nih.gov/#query=C13H8O6) |
| 6 | Methyl gallate | 7428 | [C_8_H_8_O_5_](https://pubchem.ncbi.nlm.nih.gov/#query=C8H8O5) |
| 7 | glucoside | 64689 | [C_6_H_12_O_6_](https://pubchem.ncbi.nlm.nih.gov/#query=C6H12O6) |
| 8 | Quercetin | 5280343 | [C_15_H_10_O_7_](https://pubchem.ncbi.nlm.nih.gov/#query=C15H10O7) |
| 9 | Catechin | 9064 | [C_15_H_14_O_6_](https://pubchem.ncbi.nlm.nih.gov/#query=C15H14O6) |
| 10 | Ellagic acid | 5281855 | [C_14_H_6_O_8_](https://pubchem.ncbi.nlm.nih.gov/#query=C14H6O8) |
| 11 | Isoquercitrin | 5280804 | [C_21_H_20_O_12_](https://pubchem.ncbi.nlm.nih.gov/#query=C21H20O12) |
| 12 | Mangiferin | 5281647 | [C_19_H_18_O_11_](https://pubchem.ncbi.nlm.nih.gov/#query=C19H18O11) |
| 13 | Homomangiferin | 5491388 | [C_20_H_20_O_11_](https://pubchem.ncbi.nlm.nih.gov/#query=C20H20O11) |
| 14 | Hyperin | 5281643 | [C_21_H_20_O_12_](https://pubchem.ncbi.nlm.nih.gov/#query=C21H20O12) |
| 15 | Kaempferol 3-O-rutinoside | 102180242 | [C_39_H_50_O_24_](https://pubchem.ncbi.nlm.nih.gov/#query=C39H50O24) |
| D-1 | Abemaciclib | 46220502 | [C_27_H_32_F_2_N_8_](https://pubchem.ncbi.nlm.nih.gov/#query=C27H32F2N8) |
